# EEG-Based BCIs on Motor Imagery Paradigm Using Wearable Technologies: A Systematic Review

**DOI:** 10.3390/s23052798

**Published:** 2023-03-03

**Authors:** Aurora Saibene, Mirko Caglioni, Silvia Corchs, Francesca Gasparini

**Affiliations:** 1 Department of Informatics, Systems and Communication, University of Milano-Bicocca, Viale Sarca 336, 20126 Milano, Italy; 2 NeuroMI, Milan Center for Neuroscience, Piazza dell’Ateneo Nuovo 1, 20126 Milano, Italy; 3 Department of Theoretical and Applied Sciences, University of Insubria, Via J. H. Dunant 3, 21100 Varese, Italy

**Keywords:** electroencephalogram (EEG), brain–computer interface (BCI), motor imagery (MI), wearable devices

## Abstract

In recent decades, the automatic recognition and interpretation of brain waves acquired by electroencephalographic (EEG) technologies have undergone remarkable growth, leading to a consequent rapid development of brain–computer interfaces (BCIs). EEG-based BCIs are non-invasive systems that allow communication between a human being and an external device interpreting brain activity directly. Thanks to the advances in neurotechnologies, and especially in the field of wearable devices, BCIs are now also employed outside medical and clinical applications. Within this context, this paper proposes a systematic review of EEG-based BCIs, focusing on one of the most promising paradigms based on motor imagery (MI) and limiting the analysis to applications that adopt wearable devices. This review aims to evaluate the maturity levels of these systems, both from the technological and computational points of view. The selection of papers has been performed following the Preferred Reporting Items for Systematic Reviews and Meta-Analyses (PRISMA), leading to 84 publications considered in the last ten years (from 2012 to 2022). Besides technological and computational aspects, this review also aims to systematically list experimental paradigms and available datasets in order to identify benchmarks and guidelines for the development of new applications and computational models.

## 1. Introduction

From the pioneering work of Hans Berger, which recorded the first human electroencephalographic signal (EEG) in 1924 [1], the research devoted to detecting and analyzing brain waves has increased exponentially over the years, especially in medical contexts for both diagnostic and health care applications.

The automatic recognition and interpretation of brain waves permit the development of systems that allow subjects to interact and control devices through brain signals and thus provide new forms for human–machine interactions through systems called brain–computer interfaces (BCIs).

Several applications have been developed, especially for assistive and rehabilitative purposes [2]. However, in recent decades, the rapid development of neurotechnologies, particularly wearable devices, has opened new perspectives and applications outside the medical field, including education, entertainment, civil, industrial, and military fields [3]. Among the different BCI paradigms, motor imagery (MI) deserves particular attention, given that it can be used for a variety of applications and knowing that the research community has achieved promising results in terms of performance [4].

Starting from these premises, this paper systematically reviews EEG-based MI-BCIs by following the Preferred Reporting Items for Systematic Reviews and Meta-Analyses (PRISMA) suggestions [5].

The main research question of this review paper is as follows:

RQ: *Are wearable technologies mature for EEG-based MI-BCI applications in uncontrolled environments?*

To properly answer this question, four different sub-questions are considered as follows:

1. RQ1: Is there a significant amount of EEG-based MI-BCI studies using wearable technologies in the literature that implies a promising future development of this research field, especially in uncontrolled environments and outside the medical and clinical settings?
2. RQ2: Are there common pipelines of processing that can be adopted from signal acquisition to feedback generation?
3. RQ3: Are there consolidated experimental paradigms for wearable EEG-based MI-BCI applications?
4. RQ4: Are there datasets available for the research community to properly compare classification models and data analysis?

To face these questions, the work is structured as follows. Section 2 describes how the 84 papers for the proposed systematic review have been selected, following the PRISMA suggestions. Section 3 reports basic knowledge concepts of electroencephalographic signals, brain–computer interfaces, motor imagery, and wearable technologies to provide a proper background for the comprehension of the next sections. To properly identify the contribution of this work, an overview of other survey articles present in the literature and concerning BCI systems is reported in Section 4. Section 5 is the core of this review paper, reporting systematically the motor imagery brain–computer interface wearable systems found in the state of the art, in terms of applications and employed technologies. A detailed description of signal processing, feature engineering, classification, and data analysis is also reported with the description of all the datasets and experimental paradigms adopted. Particular attention has been given in some sections to those papers that can be reproducible, either because the analyzed dataset is available, or because the computational models adopted are described with proper technical details. All information gathered from the 84 publications considered will be made available as supplementary material, organized in a detailed table (Table S1).

Finally, the answers to the presented research questions have been provided in Section 6, taking into consideration the detailed analyses of the different aspects discussed in the previous sections. Conclusions and future perspectives are presented in Section 7.

## 2. Systematic Review Search Method

The Preferred Reporting Items for Systematic Reviews and Meta-Analyses (PRISMA) [5] was employed to conduct this systematic review.

### 2.1. Eligibility Criteria

The papers included in the systematic review needed to present studies on EEG-based BCIs considering motor imagery paradigms and the usage of wearable technologies for signal acquisition. Moreover, the following inclusion criteria were applied:

- Studies published in the last 10 years (from 1 January 2012 to 22 June 2022);
- Studies published as journal articles, conference proceedings, and dataset reports.

Papers falling in the following criteria were excluded:

- Non-English articles;
- Studies published as meeting abstracts, book chapters, posters, reviews, and Master’s and PhD dissertations.

Following these criteria, studies were initially identified by the search engines defined in Section 2.2 by applying their filters, when present. Afterward, a manual scanning was conducted on titles, keywords, abstracts, and data description sections of the publications by dividing the studies extracted from the search engines equally among the review authors.

### 2.2. Information Sources

Seven search engines have been used to collect works on EEG-based BCIs concerning motor imagery paradigms and wearable technologies: IEEE Xplore, Mendeley, PubMed, ScienceOPEN, Sematic Scholar, Scopus, and Web of Science.

Google Scholar was also consulted to retrieve more information on some of the publications outputted from the other search engines and papers that were already stored in private repositories of the different research groups. For the final set of reviewed papers, the last consultation date will be reported for completeness. Table 1 reports information for each search engine specifying the authors (with initials) that used it and its last consultation date.

### 2.3. Search Strategy

The works included for manual screening were the outcome of the search conducted by querying the information sources described in Section 2.2 with the following keywords: *(EEG OR eeg OR electroencephalographic OR electroencephalography) AND (BCI OR brain–computer interface OR bci) AND (wearable OR wireless) AND (motor imagery OR motor-imagery OR MI)*.

However, before considering this final group of keywords and to understand the interest of the research community on the specific topic of EEG-based MI-BCIs with wearable technologies, we restricted the queries to a combination of the provided keywords, obtaining the results reported in Table 2. Notice that besides the first field identifying the search engines, the others report the main keywords of the considered subsets.

Notice that Google Scholar is not reported, being used only to retrieve information on papers present in private repositories or additional details on the scraped ones.

Observing the table and making a comparison between the refined searches with respect to the more general one on EEG and BCI-related papers, it appears that

- A mean (std) of 4.76% (4.98%) works of the *EEG and BCI* search are present in the *EEG and BCI and wearable* field;
- A mean (std) of 26.01% (9.38%) works of the *EEG and BCI* search are present in the *EEG and BCI and MI* field;
- A mean (std) of 0.71% (0.65%) works of the *EEG and BCI* search are present in the *EEG and BCI and wearable and MI* field, the target of the present survey.

Therefore, the interest in EEG-based MI-BCIs with wearable technologies has yet to have broader dissemination in the research community. Wearable technologies seem to be discussed, while the field of MI is extremely prolific.

All the queries followed the eligibility criteria described in Section 2.1, but where not searched for duplicates and manually screened. These procedures were applied only to the last target query.

### 2.4. Search Outcome

Following the previously described search strategies and filtering the results according to the established inclusion and exclusion criteria, 84 papers were included in the present review.

**Figure 1:**
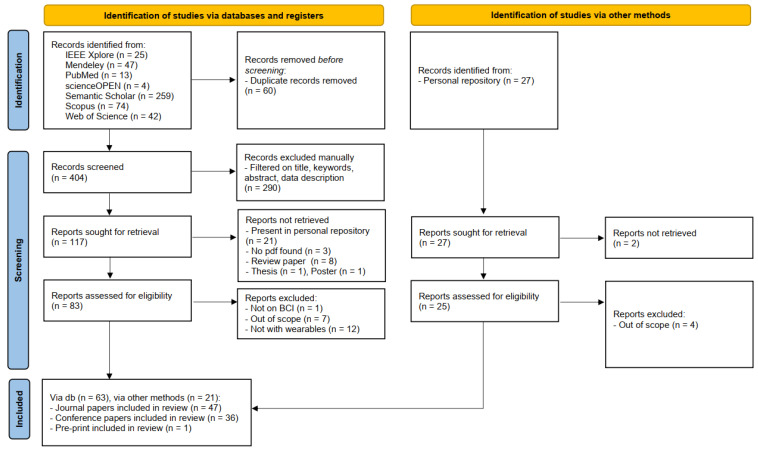
Flow diagram obtained by following the PRISMA guidelines.

Figure 1 depicts the flow diagram obtained by following the PRISMA guidelines. Notice that the diagram is divided into two main sections. The first concerns the works retrieved through the search engines listed in Section 2.2, while the second pertains to the studies in the research group’s personal repositories.

Duplicates were removed from the papers outputted by the search engines before the screening. Afterward, abstracts were manually checked by the authors ensuring that they present references to the keywords reported in Section 2.3. The number of abstracts to check were equally distributed among the authors. Reports were not retrieved if they were (i) already present in the personal repositories, (ii) not available online, or (iii) they were review papers, posters, or thesis. Finally, the papers were excluded after a thorough reading of the works if unrelated to the BCI field and/or not presenting analyses on wearable devices.

Notice that each paper was read by at least two authors.

**Figure 2:**
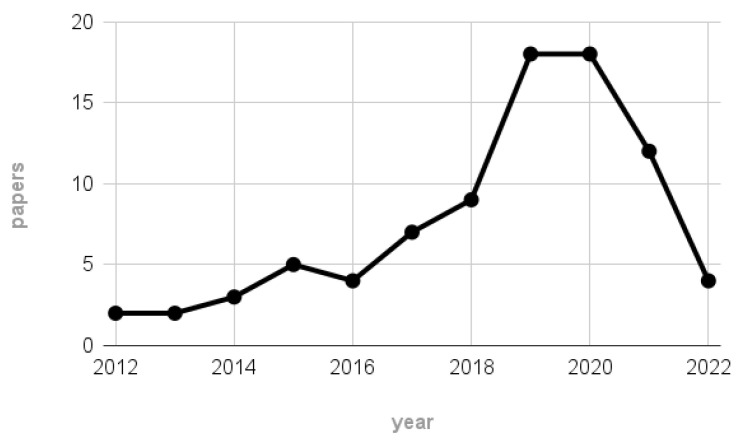
Number of papers remaining after the scraping process per each considered year (1 January 2012–22 June 2022).

For completeness, Figure 2 depicts the number of papers remaining after the screening process per each considered year. According to the plot, most of the 84 works were published between 2019 and 2020, highlighting the rise in interest of the EEG community towards MI-BCI systems employing wearable technologies and thus further justifying our interest in the reviewed topic.

Notice that the scarce number of papers related to the 2022 label may not be representative of the hypothesized publication trend, since the scraping on 2022 publications stopped on 22 June.

Detailed notes on the reviewed papers are included in Table S1 as supplementary material to the present review. The notes are organized to provide a clear reference to the papers and to follow the core section of this review (Section 5), analyzing (i) field of applications, (ii) employed technologies, (iii) signal processing and analysis methodologies, (iv) BCI feedback, and (v) dataset information.

## 3. Background Information

This section is devoted to the explanation of some basic knowledge related to the target of this survey. Therefore, an overview of EEG signals, BCIs, MI, and wearable technologies is provided.

### 3.1. Electroencephalogram

**Figure 3:**
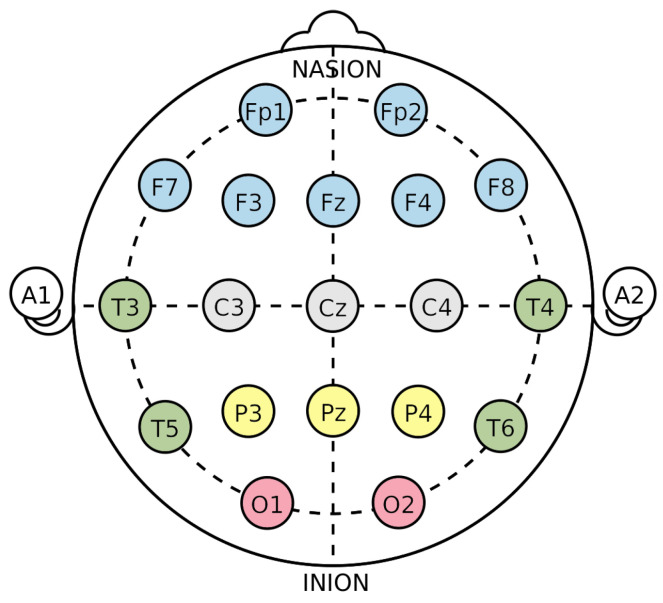
10-20 International system (adapted from https://commons.wikimedia.org/w/index.php?curid=10489987 accessed on 31 January 2023). The letter correspondences are as follows: frontopolar (Fp), frontal (F), central (C), parietal (P), occipital (O), temporal (T). Auricular (A) electrodes are also included.

**Figure 4:**
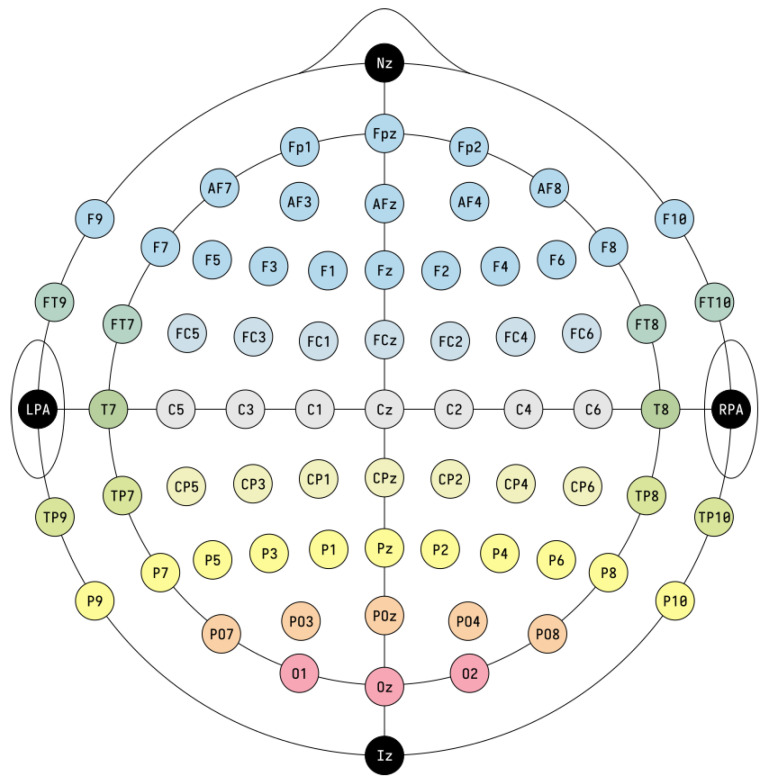
10-10 International system (adapted from https://commons.wikimedia.org/w/index.php?curid=96859272 accessed on 31 January 2023). The letter correspondences are as follows: frontopolar (Fp), AF between Fp and F, frontal (F), FC between F and C, central (C), CP between C and P, parietal (P), PO between P and O, occipital (O), temporal (T), FT between F and T, TP between T and P. The system also presents the nasion (Nz), inion (Iz), left and right pre-auricular point (LPA and RPA).

In this review, the considered applications are based on the use of the electroencephalogram (EEG) as a non-invasive technology to acquire brain signals. In fact, the EEG is able to record the brain’s electric potentials deriving from the activation of the cerebral cortex in response to neural processes [6], which could be spontaneous or evoked by external stimuli [7]. The resulting signal is a time series characterized by time, frequency (Section 3.1.1), and spatial information, and acquired with non-invasive sensors (called electrodes or channels) placed on the scalp of a subject [8,9,10,11]. Moreover, the electrode positioning usually follows standard placement systems, which define the distance between adjacent electrodes taking into consideration specific anatomical landmarks, such as the distance from the nasion to the inion [12]. The most commonly used systems are the 10-20 and 10-10 International ones [12,13], which are depicted in Figure 3 and Figure 4. Notice that the electrodes are identified by letters, which refer to the brain area pertaining to a specific electrode, and a number (odd for the left hemisphere, even for the right one). The midline placement is identified by a *z*.

Therefore, the sensors are usually placed following standard locations that respect the brain’s anatomical structures. However, it could happen that the neural activations are not recorded uniformly or have irregular samples [14], and that the volume conduction effect may provide indirect and imprecise recordings [15].

Moreover, the EEG signals are [16] easily affected by noise, and thus usually have low Signal-to-Noise Ratio (SNR) [7,17].

EEG data are also heterogeneous,

- being non-stationary signals varying across time [18];
- being subject-specific, due to the natural physiological differences between subjects [16];
- varying in the same subject depending on their physiological and psychological conditions, and changing from trial to trial [19];
- being influenced by experimental protocols and environmental conditions [7,16].

Therefore, coupling the EEG signals with specific brain activities is a difficult and ambiguous task [20].

In the following, details on the frequency information characterizing the EEG signals and the noise affecting them will be given to provide a better overview of these peculiar data.

#### 3.1.1. EEG Rhythms

As previously introduced, the EEG signal is characterized by frequency information, which is provided by different frequency bands, called rhythms, associated with specific brain activities and functions [21]. Table 3 provides a brief overview of the EEG rhythms and their frequency ranges.

Starting from the lowest frequency band, the delta (δ) rhythm presents slow waves typical of infants or predominant during deep sleep [12,22]. The theta (θ) rhythm is instead elicited by emotional stress and is present in sleepy adults [12,21,22]. During a relaxed but awake state, the alpha (α) rhythm is elicited [21]. Notice that α and mu (μ) rhythms share similar frequency components, but μ is related to the motor cortex functionalities [22] and is usually non-sinusoidal [23]. The influence of the μ band on motor imagery tasks will be discussed in detail in Section 3.2.

Concerning the beta (β) rhythm, it is present in alert states due to active thinking or attention and may also be an effect of anxiety [12,21]. Finally, the gamma (γ) rhythm is intertwined with more complex cognitive functions, such as sensory stimulus recognition and two senses combination [12,22].

Knowing the EEG intrinsic frequency characteristics, it is possible to exploit them to design specific experimental paradigms and make assumptions on EEG data analyses.

### 3.2. Motor Imagery

Motor imagery (MI) is the imagined rehearsal of an actual movement [24,25]. During motor imagination, a person seems to consciously access motor representation, i.e., the intention of making a movement and a preparation for it, which is usually an operation performed unconsciously [26,27].

Moreover, the imagination can be performed using a first (internal) or third (external) person perspective [28]. In the first case, the person should feel like they are performing the imagined movement; in the external perspective, the person should feel like watching themselves while performing the movement [28].

Furthermore, numerous research studies have found that motor imagery and representation present the same functional relationships and mechanisms [23,26,29,30], which results in the activation of the same brain areas when performing the actual movement and imagining it [27,31,32].

In fact, analyzing the EEG signals recorded from the primary sensorimotor cortex during the executed or imagined movement of specific body parts, variations in amplitude, mainly of the μ and β rhythms, can be observed [29,30]. These non-phase-locked-to-the-event variations are called Event-Related Desynchronization and Synchronization (ERD/ERS), corresponding to a decrease or increase in the rhythm’s amplitude, respectively, [33].

Before performing a movement, the μ and β rhythms are subject to an ERD [34,35]. Instead, the deactivation of the motor cortex due to movement stopping elicits an ERS on the β frequency band [36].

These pieces of evidence justify the supporting role of MI training in motor execution improvement [27] and generally in the enhancement of neuroplasticity [24,37], i.e., the brain’s ability to change in response to new conditions [38]. Moreover, the MI-related phenomena can be easily exploited to provide an MI-based control of a BCI system [34].

However, the MI ability is subjective due to each individual difference, and thus needs to be assessed before being exploited in experiments/applications, or to be trained [28,31]. Particularly in the field of MI-based BCIs, proper motor imagery task completion may require a long time, and Kaiser et al. [39] affirm that a good BCI control is usually achieved when the subject is at least able to perform 70% of the required tasks accurately.

### 3.3. Brain–Computer Interfaces

In recent years, neurotechnologies faced great developments and have brought important solutions in the collection and analysis of physiological data in several fields. In particular, they allowed an advance in the identification and treatment of neurological diseases [40] in the medical sector.

Starting from these premises, Brain–Computer Interfaces (BCIs) were born and progressed to provide online brain–machine communication systems [41] that allow the control of devices or applications by recording and analyzing brain waves.

**Figure 5:**
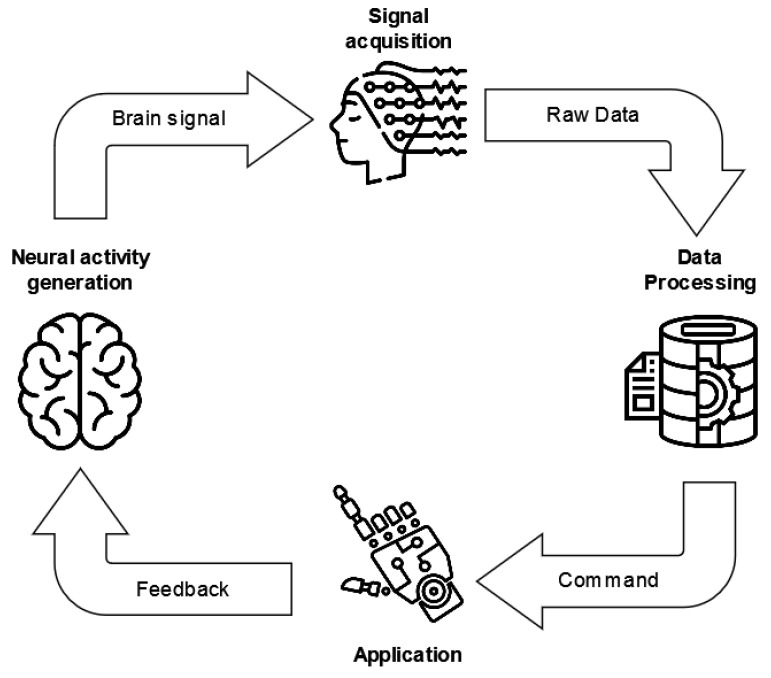
BCI system standard life cycle. The three main modules are reported, i.e., the signal acquisition, data processing, and application modules.

The life cycle of current BCI systems is based on a standard architecture, defined by three main modules (Figure 5): the signal acquisition, processing, and application modules [42].

The *signal acquisition module* deals with the input of the BCI system and thus is responsible for recording the physiological signals, which are amplified and digitized. Afterward, these data become the inputs of the *data processing module*, which processes the signal in order to convert it into commands for an external device or application. Moreover, this module mainly performs (i) signal preprocessing, with the aim of increasing the signal-to-noise ratio [43] by removing artifacts; (ii) feature engineering to extract characterizing and significant information from the signal; and (iii) classification to translate the signals and their features into machine-readable commands.

Finally, the *application module* provides feedback to the BCI user.

Considering all the EEG-based BCI applications, distinctions can be made among motor imagery (described in Section 3.2), external stimulation paradigm, Error-Related Potentials, inner speech recognition, and hybrid paradigms [44,45].

The external stimulation paradigm refers to the fact that brain waves can be modified by external auditory, visual or somatosensory stimuli. Examples of this paradigm are the Event-Related Potential (ERP) and the steady-state visual evoked potential (SSVEP). ERPs are characterized by components that identify a certain shape of the signal wave. One of the most studied ERPs in the BCI field is the P300, a component related to a positive signal deflection that creates a peak starting at 300 ms when an unexpected stimulus appears [46].

The SSVEP is instead a phenomenon consisting of a periodic component of the same frequency that occurs when a subject looks at a light source that flickers at a constant frequency. This occurs at frequencies in the range of 5–20 Hz and is mainly detectable in the occipital and temporal lobes [47]. The SSVEP paradigm has a great advantage: since the relative stimuli are exogenous, it is a no-training paradigm and thus should not require long training times. However, SSVEP may lead to fatigue or trigger epileptic seizures [44], and thus, enhancements to the BCI systems based on this paradigm should be developed [48].

When an error or a mismatch arises between the user performing a task and the response provided by the BCI system, an Error-Related Potential occurs (ErrP). ErrPs are potentials that represent the user’s perception of an occurrence of an error in the BCI system. ErrPs are reliably detected on a single-trial basis, and thus have immense potential in real-time BCI applications [49].

Moreover, new paradigms are progressively appearing in the literature concerning inner speech recognition, which is devoted to the identification of an internalized process where a person thinks in pure meanings, generally associated with auditory imagery of their own inner voice [45].

Even thought systems based on these paradigms seem to have a great number of advantages, BCI systems usually present some limitations, and thus, hybrid BCIs could be considered to overcome them [50]. Hybrid paradigms are usually employed by exploiting two or more of the described paradigms. In fact, as demonstrated by several studies [51,52,53], the combination of two or more paradigms can lead to a significant improvement in the performance of the BCI system. For example, in [51], the authors combine P300 and SSVEP to create a high-speed speller BCI system with more than 100 command codes.

As previously stated, EEG-based BCI systems are born for medical purposes, from neurorehabilitation to prevention, up to the identification of pathologies and diagnoses. For example, EEG signals are used for the identification and prevention of epileptic seizures, and systems have been developed allowing their high detection and prediction accuracy, as well as a better localization of epileptic foci [40]. BCI systems are also used in the neurorehabilitation field, for example, for the treatment of patients who suffer from motor disabilities after a stroke [54,55,56] or who are affected by Parkinson’s disease [57,58]. In particular, motor imagery-based BCIs have proven to be an effective tool for post-stroke rehabilitation therapy through the use of different MI-BCI strategies, such as functional electric stimulation, robotics assistance, and hybrid virtual reality-based models [55]. BCIs are also used to detect health issues such as tumors, seizure disorders, sleep disorders, dyslexia, and brain swelling such as encephalitis [59].

In addition to these medical and neurological rehabilitation uses, there are many other fields of application of BCIs [59,60,61,62].

For example, the marketing sector uses EEG data to evaluate advertisements in terms of consumer attention and memory [59].

Moreover, the study of brain signals in the field of education has provided more insights into the degree of studied information and how the studying experience could be tailored to a single student. This could provide improvement in students’ skills in real-life scenarios, instead of focusing only on information memorization. Moreover, the students could better develop competencies such as adaptive thinking, sense making, design mindset, transdisciplinary approaches, and computational skills [63].

Further development of BCI systems is also represented by their integration into the world of the Internet of Things. In fact, BCIs could be assistants able to analyze data such as mental fatigue, levels of stress, frustration, and attention [64], while being embedded in smart devices.

Therefore, the study areas that make use of BCI systems are diverse and increasingly numerous. Outside the medical sector, EEG-based BCI technologies have been receiving more attention due to the development of non-medical wearable EEG devices. In fact, these systems guarantee access to a wide range of users, given their high portability and low cost.

Generally, BCI technologies need to go under the scrutiny of experts to provide appropriate user-centered systems and allow a proper insight into their clinical usefulness and practicality [65]. These necessities can be detrimental in terms of efficient BCI system management costs, even though the initial technologies may be sufficiently low-cost [65].

### 3.4. Wearable Technologies

As introduced in the previous Section 3.3, considering the new demands from the general public and the need to move from laboratories and research centers to in-home and real-life environments, EEG-based BCI technologies are moving to low-cost, portable, and easy-to-use for non-expert solutions.

Traditional laboratory EEG devices usually have helmet structures with holes in which the sensors (electrodes) are installed. The helmet is placed on the patient’s scalp, and each electrode is connected to the recording tool by using cables. This may translate into a very large amount of time for preparing a test. e.g., as described by Fiedler et al. [66] usually a 256-channel device requires long times to connect each electrode to the registration system and for patient preparation. This also makes it impossible for the patient to wear the device at any time of the day at home or anywhere else. Fiedler et al. [66] also propose a 256 dry electrode cap that counteracts these issues.

Starting from this awareness and to overcome the problem of the discomfort of wired instruments, EEG wearable models were born. Wearable devices represent an evolution of the classic EEG tools. They are smaller devices that can be used potentially in any place and at any time. In fact, Casson et al. [67] defined wearable devices as follows:

*the evolution of ambulatory EEG units from the bulky, limited lifetime devices available today to small devices present only on the head that can record EEG for days, weeks, or months at a time*.

By removing the cumbersome recording units and connecting wires and replacing them with microchip electrodes with embedded amplifiers, quantizers, and wireless transmitters, it was possible to achieve the goal of creating devices that recorded and sent data wirelessly.

Although wired EEGs are more stable and can transmit more data in less time [68], wearable devices are more comfortable and can be used wherever, thanks to their portability. Moreover, it is possible to avoid the artifacts caused by the movement of cables and electrodes of wired EEG by using wearable EEG devices.

Notice that the electrodes of a wearable device need an adequate electrical connection with the scalp of the subject. The currently available devices are equipped with three types of electrodes: dry, wet (gel-based or saline solution-based), and semi-dry. Dry electrodes of an EEG do not need to use a gel nor a saline solution to be connected to the scalp. This implies a greater simplicity of positioning, also reducing the setup time at the expense of good signal conduction. Wet sensors can be divided into two categories: gel-based electrodes or saline solution-based electrodes. Gel-based electrodes make use of an electroconductive gel applied on each electrode. Gels generally reduce movement and skin surface artifacts by creating a more stable conductive connection between the electrode and the scalp compared to saline solutions [69]. However, the time for preparing the subject could increase, and the subject may be uncomfortable due to the gel residues on the hairs and scalp. The cap and electrodes should also be carefully cleaned after usage.

On the other hand, some devices have electrodes imbued with a conductive saline solution in order to help make low-impedance electrical contact between the skin and the sensors [68].

It is also important to consider that wet electrodes-based devices have some limitations [69]: the skin must be prepared to reduce the impedance of the scalp, and the procedure can be annoying or sometimes painful. Moreover, if too much gel is used, it could affect signal transmission by causing a short circuit between two adjacent electrodes.

On the opposite, semi-dry electrodes require only a small amount of electrolyte fluid, combining the advantages of both wet and dry electrodes while addressing their respective drawbacks. Their setup is as fast and convenient as their dry counterparts [70].

Even though the evolution of wearable technologies seems to be rapidly escalating, numerous issues remain to be addressed, especially if the final goal of these EEG wearable devices is to be used in any context. Firstly, the non-expert user may find it difficult to wear the electrodes correctly. As a result, the electrodes may be damaged or not correctly record the signal. A good device should guide the novice user to a fast and effective positioning on the scalp, indicating whether the contact of the electrodes is stable or not. On the other hand, it is essential to make devices smaller and less bulky, while making them more resistant to artifacts [71]. Another sore point concerns the battery life, which is still too low for the instrument to be comfortably used outside research centers. Most wearable devices have been realized for general functions and applications that lack support for signal processing and feedback generation [72], resulting in low-quality signal processing.

In light of these limitations, new designs are emerging for new generations of wearable EEG devices. Thanks to several studies showing that the EEG signal can be recorded on the forehead [73] and behind the ears [74,75], tattoo-like devices are emerging. They offer better wearability and versatility [76]. In fact, they can be connected directly to the skin through adhesive materials without the use of gels or external supports. With the advantage of having features such as ultra-thin thickness, ultra-softness, and high adherence to the skin, the electrodes can comply with skin deformation, providing a stable signal quality [77]. The downside of using such devices is that they may not be able to cover all areas of interest, such as hair-covered areas, making them unsuitable for some uses of EEG monitoring.

To access more details on the evolution of hardware components, we refer the readers to [71,78].

## 4. Overview of Survey Articles on EEG-Based BCIs

In this section, an overview of survey articles present in the literature and concerning BCI systems is reported to provide a general assessment of the topics related but not superimposed to the target of the present paper.

In fact, most of the analyzed surveys address different application research areas and experimental paradigms. None of them focus exclusively on MI tasks, except the works by Palumbo et al. (2021) [79], who consider MI tasks in the sole field of wheelchair control, and the review study by Al-Saegh et al. (2021) [4], that concerns the use of deep neural networks in the context of MI EEG-based BCIs.

In particular, Palumbo et al. [79] provide a systematic survey of EEG-based BCIs for wheelchair control through motor imagination by including 16 papers published since 2010. The authors focused on (i) the MI paradigms and the type of commands provided to move the wheelchair, (ii) the employed EEG system (presenting sensors for other biomedical signal recording and the number of positioned electrodes) and the wheelchair components, and (iii) the EEG signal management procedures. The authors want to especially provide a clear assessment of the limitations of current biomedical devices when the end user is affected by any kind of motor disability. Moreover, they point out the main challenges arising when having to face the development of an efficient and reliable BCI to control a wheelchair. Firstly, multiple commands are required to allow correct control of the wheelchair, and thus a multi-objective problem characterizes the system. However, adding more commands may affect the performance of the BCI both in terms of accuracy and time consumption. Secondly, the BCI performance is ultimately dependent on the user, who may fail to perform the MI tasks. Finally, wheelchair control requires constant concentration on the task and thus increases the users’ mental workload.

Considering instead the work of Al-Saegh et al. [4], the use of deep neural networks in the context of EEG-based MI-BCIs is surveyed. The authors retrieved 40 papers published between 1 January 2015 and 31 March 2020. An analysis of the employed datasets was performed, and the authors found information on 15 datasets, of which 7 are publicly available. They notice that they vary significantly in terms of electrodes, subjects, number of MI tasks, and trials. However, most of the datasets seem to rely on experimental paradigms concerning the MI of the right/left hand, feet, and tongue. Moreover, the authors provide an assessment of the most used frequency ranges, extracted features, deep network architectures, and input formulations, highlighting the variety of deep learning (DL) model designs.

In what follows, the review articles are reported according to their topics (experimental paradigms and applications, technological aspects, signal processing, and analyses) and in chronological order of publication.

Considering experimental paradigms and applications, a comprehensive survey of different BCI experimental paradigms can be found in [44], published in 2019.

A general survey on EEG technologies and their application is instead presented by Soufineyestani et al. [68] (2020).

Moving to the technological aspects, a brief review of wearable technologies for smart environments is presented in [80], where the authors dedicate two sections to devices and applications for BCI systems. Considering these last topics, the technologies available at the time of the review (2016) are precisely listed, and advancements in EEG devices are easily detectable, especially considering the use of dry sensors and the presence of products for the general public. This information provides a good starting point to compare wearable technologies.

Instead, a detailed overview of the hardware components of wearable EEG devices was provided by [71] in 2019.

The survey by TajDini et al. [81] (2020) focuses on wireless sensors and, in particular, on the assessment of consumer-grade EEG devices for non-medical research. The authors compare 18 products in terms of sensor type (dry, wet, semi-dry), number of channels, sampling rate, accessibility to raw data, operation time, and price. The analysis of the literature is explored based on the different application domains: cognition (emotion recognition and classification, attention, mental workload, memory), BCI (ERP, SSVEP, MI, and other), educational research, and gaming.

The review by Portillo-Lara et al. [82] (2021) starts with an overview of the neurophysiological mechanisms that underlie the generation of EEG signals and then focuses on the state-of-the-art technologies and applications of EEG-based BCIs. Different electrode interfaces and EEG platforms are analyzed and compared in terms of electrode type and density, functionality, portability, and device performance. The advantages and disadvantages of different electrode designs are enumerated. The technical specifications of 18 commercially available EEG platforms are also compared in terms of electrodes, channel count, sampling rate, weight, battery life, resolution, and price. Both medical and non-medical uses are reviewed in the article.

Instead, the review by Jamil et al. [83] (2021) aims to identify the main application areas that use EEG-based BCI devices and the most common types of EEG-based equipment, considering both wired and wireless devices. They present a systematic review using four search engines (PubMed, IEEE, Scopus, and ScienceDirect). The search strings used were (BCI OR Brain–computer interface OR BMI OR brain–machine interface) AND (EEG OR electroencephalogram) AND (rehab* OR assist* OR adapt*). The inclusion criteria were limited to publication years 2016–2020. After the screening, 238 articles were selected and classified according to the following four research areas: education, engineering, entertainment, and medicine. They found that the medical area is the most frequently used (80%). Wired devices were used in the studies by 121/238 articles, while the remaining 117 reviewed manuscripts employed wireless technologies.

Concerning signal processing and analyses, the feature extraction techniques widely used in the literature were reviewed in 2019 by Aggarwal et al. [43]. Moreover, a survey on EEG data collection and management (processing, feature exaction, and classification) is provided by Reaves et al. [40] (2021), considering 48 papers from high-impact journals. The authors also include lists of devices and publicly available datasets.

A comprehensive review was presented by Gu et al. [84] in 2021. The authors provide a broad overview of BCI systems and their application areas. Moreover, they make a general presentation of invasive, partially, and non-invasive brain imaging techniques and subsequently focus on EEG-based BCIs. Their review is organized to provide a consistent survey on (i) advances in sensors/sensing technologies, (ii) signal enhancement and real-time processing, (iii) machine learning (especially transfer learning and fuzzy models) and deep learning algorithms for BCI applications, and (iv) evolution of healthcare systems and applications in BCIs (e.g., concerning epilepsy, Parkinson’s/Alzheimer’s disease, and neurorehabilitation). Their analysis was performed on about 200 papers, considering publication years between 2015 and 2019.

## 5. EEG-Based MI-BCIs through Wearable Systems

In this section, we discuss and analyze the main issues and characteristics of the works considered for the present review. A detailed table that summarizes the collected information is made available as supplementary material (Table S1).

### 5.1. BCI Application and Feedback

As a first assessment of the reviewed papers, an overview of the applications and feedback types of these EEG-based MI-BCI systems is provided in this section.

The main aim is to better understand the wide application of BCIs and the shift of experimental paradigms due to the need for consumer-grade applications in real-life environments.

By analyzing the reviewed papers (Table S1), it is clear that the field of application and the feedback are extremely interconnected.

Moreover, motor imagery seems to be particularly used to provide control for external devices, especially for rehabilitation purposes. Therefore, the feedback usually consists of movements of robots, wheelchairs, prostheses, drones, and exoskeletons.

There are cases in which the feedback is visually provided on a monitor or exploiting virtual reality systems [85].

Moreover, feedback can help in modulating the MI ability of users. For instance, Jiang et al. [86] deal with the creation of a BCI system that uses discrete and continuous feedback in order to improve practicability and training efficiency. The results show that continuous feedback successfully improves imagery ability and decreases the control time.

Starting an in-depth analysis of the application fields, 30 of 84 research studies have medical purposes [87,88,89,90,91,92,93,94,95,96,97,98,99,100,101,102,103,104,105,106,107,108,109,110,111,112,113,114,115,115].

Considering the stroke rehabilitation field, Mattia et al. [87] built a BCI system to enhance post-stroke hand functional motor recovery by using ecological feedback, i.e., the visual representation of the patient’s hands, as a feedback tool for the user. A BCI system for recovering people who suffer from post-stroke hand disability has also been proposed by [116]. Firstly, an offline analysis was performed. Afterward, the online system was initially tested on healthy subjects and then on 10 stroke patients affected by a hand disability. The subjects received virtual feedback by controlling a virtual exoskeleton.

Another post-stroke rehabilitation BCI system is presented by Barria et al. [88], who propose a paradigm consisting of motor imagination with visual stimulation and motor imagination with visual-haptic inducement to control an ankle exoskeleton.

Moving to other motor disorders, [89] assumes that people with motor disorders need the help of a caregiver to start a BCI. Therefore, they aim to identify movement intention to initiate BCI systems. Instead, [90] examined neurofeedback manipulation to ensure self-regulation of brain activity as a potential treatment for post-traumatic stress disorder, considering both offline and online analyses. The feedback was shown through a video game, and the participants were asked to manipulate their brain activity to control the game.

Finally, Looned et al. [91] demonstrated the feasibility of a system that assists individuals with neurological disorders to, for example, drink a glass of water independently. The application of this system is implemented through the movement of a robotic arm that assists the movement of the human arm.

Li et al. [92] created a hybrid BCI system to control a lower extremity exoskeleton. By combining EEG data with electromyographic data, they developed an exoskeleton able to help subjects while climbing stairs.

BCI applications are also developed in the entertainment, game, and device (vehicle, robots) control areas [117,118,119,120,121,122,123,124,125,126,127,128].

In particular, [117] focused on the BCI performance in a competitive multi-user condition. In fact, users had to control a humanoid robot in a race against an AI. The authors believed that the game conditions could help the users maintain high motivation and thus increase the effectiveness of the BCI system. However, they found out that there is no significant difference between competitive multi-user conditions and single-user conditions.

Device control is also proposed in [118]. A robotic quadcopter was intended to be controlled in three-dimensional physical space by a BCI user. After a training phase with a virtual drone, subjects modulated their sensorimotor rhythms and controlled a physical drone. Visual feedback was provided via a forward-facing camera on the hull of the drone.

Instead, Alanis et al. [119] created an immersive BCI system. In fact, the users could control the movement of a humanoid robot in a first-person perspective, as if the movement of the robot was their own.

Another application is the one presented by Xu et al. [120], who proposed a motor imagery EEG-based continuous teleoperation robot control system with tactile feedback. The imagination of the user’s hand movements was translated into a continuous two-dimensional control signal and transmitted to the remote robotic arm (using the TCP/IP protocol), allowing it to move remotely through a wireless connection. The user received feedback through a vibrotactile stimulus based on the tactile information of the robotic arm. The authors demonstrated that vibrotactile stimulation can improve operator telepresence and task performance.

**Figure 6:**
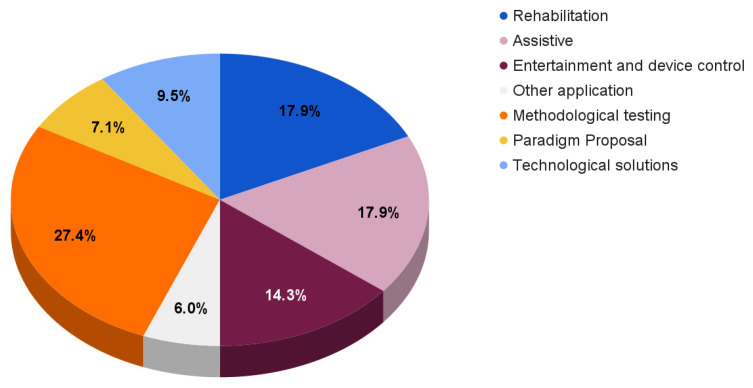
Reviewed paper distribution according to different fields of applications and aims.

For completeness, Figure 6 reports the reviewed paper distribution according to different fields of application and aims.

With respect to the previously discussed solutions, the reported papers mainly focus on rehabilitation systems (17.9%), assistive BCIs (17.9%), and entertainment and device control solutions (14.3%).

The remaining works provide

- Methodological testing (27.4%), which includes new frameworks and processing techniques [113,129,130,131,132,133,134,135,136,137,138,139,140,141,142,143,144,145,146,147,148,149,150];
- Paradigm proposals (7.1%) focusing on experimental paradigm descriptions and their possible comparison with other paradigms [151,152,153,154,155,156];
- Technological solutions (9.5%), which include innovative designs concerning electrodes, amplifiers, or EEG devices [86,157,158,159,160,161,162,163].

### 5.2. Employed Technologies

This section is devoted to the revision of the technologies employed by the reviewed papers. Particular attention is given to EEG-related devices, which are one of the main focuses of this review and of which some information is reported in Table 4.

Notice that the first (*Device*) column of Table 4 provides the product names and presents an asterisk (*) when the devices are amplifiers (BrainMaster Discovery 24E g.USBamp, g.Nautilus Multi-Purpose, NuAmps, Synamps 2/RT) or data acquisition (Cyton Biosensing Board) tools. The link to the product is also reported as a citation. The producers are instead presented in the second column, and a good number of devices from Emotiv can be detected. Three g.Nautilus entries are reported due to the absence of a clear indication of the devices used by the related works [89,90,105,125,130,145,146,151,153,155].

The *Electrodes* field presents firstly the number of sensors or recorded channels and if they are wet (W), dry (D), or semi-dry (S) electrodes. Notice that for the Starstim device, the electrode type is marked as tES-EEG, having that its sensors allow both transcranial electrical stimulation and EEG monitoring. Observe that 9 of 21 devices can be used with both wet and dry electrodes and six products do not provide clear information on the sensor types.

Additional information on device pricing (updated to 3 October 2022) is reported in column 4. The need to ask the producers for the price of the majority of the products is immediately detectable. Finally, the *Papers* field provides the list of reviewed works employing a specific device. A total of 13 of 84 papers [86,123,128,134,138,143,154,158,159,160,161,162,163] do not appear in this column, having that the authors design and use custom EEG headsets. Finally, Khan et al. [135] and Vourvopoulos et al. [162] compare their custom devices with Emotiv EPOC+ and Enobio 8, respectively.

After having given a brief overview of the synthetic information regarding EEG-related devices, it is possible to move to a deeper analysis of how these technologies have been effectively used by the authors according to the table present in the Supplementary Materials (Table S1).

A total of 14 of 84 works prefer reducing the number of electrodes provided by the device producers [88,92,104,106,110,126,142,162] and especially make a selection on the channel placed over the central cortical area. In particular, only the C{3,4} [139,147], Cz [92,143,148], and C{1,2} [102] electrodes are considered.

Moreover, some of the authors clearly specify the sampling rate used for signal acquisition that goes from 125 Hz [113], 128 Hz [91,110,122,133,135,139], 250 Hz [98,99,112,145,149], 256 Hz [135], and 500 Hz [88,92,93,97,103,117,137,142,147,148,152,171,172] to 512 Hz [124].

Finally, considering the other technologies presented in the supplementary Table S1, the massive use of Bluetooth technology for wireless communication between wearable devices and acquisition/control tools is immediately observable. Moreover, some sensors have been used together with EEG devices like the electromyogram [87,92,113,175,177], functional electrical stimulation devices [102], near-infrared spectroscopy tools [104], and magnetic resonance imaging devices [150].

Notice that feedback was also provided by considering different tools, such as exoskeletons [88,92,100,103], wheelchairs and vehicles [98,99,106,108,110,122], rehabilitation and assistive robotic tools [91,97,105,107], eye tracking devices [171], movement tracking devices [171], virtual reality devices [93,101,119], robots [114,117,125,126], and simulators [127].

### 5.3. Signal Processing and Analysis

This section is devoted to the second step of the BCI life-cycle and, in particular, to signal preprocessing, feature engineering and channel selection, and data classification and analyses.

#### 5.3.1. EEG Data Preprocessing

EEG signals contain artifacts from internal sources due to physiological activities, e.g., ocular and muscle movements, cardiac activity, respiration, and perspiration. Moreover, artifacts can be generated by external sources related to environmental and experimental noise, e.g., power line and mobile phone interference, electrode movements, and electromagnetic components [7]. Each of these types of noise has its own frequency band and can affect brain rhythms differently.

Due to the non-stationarity and non-linearity of EEG signals, it is difficult to remove these artifacts without the loss of neural information [192].

In particular, EOG artifacts (related to eye movements) and EMG artifacts (related to muscle movements) are considered among the most common sources of physiological artifacts in BCI systems [193].

In the case of wearable devices, the possibility of introducing other artifacts (e.g., data transmission fault, electrical interference), with respect to traditional wired devices, increases [194]. Therefore, the preprocessing module of a typical BCI life-cycle becomes fundamental to provide better data as inputs to the subsequent modules.

From the analysis of the literature summarized in Table S1, we observe that 60 out of 84 articles indicate some details on the preprocessing step of the BCI life cycle. From this subset, we list the main techniques adopted, enumerating only those papers that explicitly reported them:

- Nine works assumed that source signals are statistically independent of each other and instantaneously mixed, and apply Independent Component Analysis (ICA) to remove noise, mainly due to eye movements and eye blinks [93,95,103,141,147,150,152,158,171]. EEGLAB toolbox [195] is frequently employed by the authors to implement ICA;
- A total of 21 papers explicitly indicate that notch filtering is applied to eliminate the power line interference at 50/60 Hz [89,92,97,98,107,111,113,119,131,132,136,140,142,143,144,151,153,155,157,158,174];
- A number of 11 works applied temporal filtering approaches such as Butterworth of different orders and cutoffs: third order filter in 0.5–30 Hz [148] or in 4–33 Hz [123], fourth order in 16–24 Hz [88], fifth order in 8–30 Hz [96,114,132,142,143] or in 1–400 Hz [113], biquad tweaked Butterworth in 8–13 Hz [138], and sixth order in 8–30 Hz [153];
- A total of 13 papers applied spatial filtering approaches such as the Common Average Reference (CAR) filter [99,120,132,138,139,148] and Laplacian ones [88,97,101,103,140,172,177].

#### 5.3.2. Feature Engineering

The next step after the signal preprocessing is the feature engineering stage, where the relevant information about the EEG signals is extracted and analyzed.

As detailed in Section 3.2, during motor movement or imagination, μ and β rhythms are interested in a Desynchronization (ERD). Instead, the deactivation of the motor cortex goes against a Synchronization (ERS) of the β rhythm.

Therefore, the power changes due to ERD/ERS encodes relevant information on MI. In general, ERD (ERS) is observed in the contralateral (ipsilateral) sensorimotor area. Taking into account these phenomena, Common Spatial Pattern (CSP) and band power features (that represent the power of EEG signals for a given frequency band averaged over a time window) are natural choices with respect to feature extraction methods and are applied by most of the works within the field of EEG analysis. The lateralization index between hemispheres is also used to describe the asymmetry of neural activation intensity [93].

Following the analysis of Table S1 provided as supplementary material and taking into account the articles that explicitly indicate the feature extraction steps performed, we have that

- In the frequency domain, the authors of 25 papers compute the Power Spectral Density (PSD) of the signal, usually through Fast Fourier Transform (FFT) or Welch’s method [86,88,89,92,93,94,97,104,109,111,114,123,124,136,138,139,140,144,145,154,160,161,162,171,177];
- In the time–frequency domain, wavelet transform-based methods are employed in 8 studies [89,122,129,141,147,154,158,163];
- In the spatial domain, CSP-based approaches are applied in 17 articles [90,92,93,96,99,100,110,111,134,135,137,146,150,151,153,168,175]. Variations of the CSP are found in [132], which considers local mean decomposition CSP, and [146], which exploits filter bank CSP, among others;
- In the temporal domain, statistical features such as the standard deviation, skewness, kurtosis, entropy, and energy are considered [89,141,183].

Notice that most of the authors combine features from multiple domains in order to obtain a final, more robust feature vector able to improve the classification accuracy.

Nearly all the reviewed articles work with different combinations of hand-crafted features, but recently deep features extracted by convolutional neural networks (CNNs) have also been considered [110,115,126,131,143].

With respect to feature selection, different methods are applied, such as ICA [146], joint mutual information [90,133], generalized sparse discriminant analysis (that is employed to perform feature reduction and classification contemporaneously) [113,168], and sequential backward floating selection techniques [135].

To reduce the data dimensionality, the traditional channel set C{3,4,z}, located over the central cortical area, is often considered [92,147,148,158,160,175]. However, in several cases, the choice of the electrodes mainly depends on the used device [86,109,112,141,144]. Electrodes positioned over the fronto-central and central-parietal areas are also frequently taken into account. Electrode subsets are also added to the C{3,4,z} group: C{1,2} [102,142], FC{3,4} [154], and Fpz and Pz [162]. Daeglau et al. [117] selected the electrode set constituted by Cz and CP{1,z,2}.

Moreover, different groups of eight electrodes are selected by some of the reported studies: FC{1,2} + C{3,z,4} + CP{1,2} + Pz [98], F{3,4} + C{3,z,4} + T{7,8} + Pz [97], C{1,3,z} + CP{1,5} + FC{1,5} + P3 [171], FC{5,6} + C{1,2,3,4} + CP{5,6} [93], and Fp{1,2} + Fz + C{z,3,4} + O{1,2} [157]. Instead, groups of 9 electrodes have been considered by [103,140] (C{z,1,2,3,4} + CP{1,2} + FC{1,2}), while 10 electrodes have been selected by [101] (C{1,2,3,4,5,6} + FC{3,4} + CP{3,4}).

#### 5.3.3. Classification and Data Analysis

As a final processing step, classification and data analysis are performed to provide a specific assessment of the EEG data or to allow the correct feedback execution.

Concerning the reviewed papers, notice that the main strategies employed can be divided into (i) traditional Machine Learning (ML) models, (ii) DL architectures, (iii) other supervised learning techniques considering ensemble and transfer learning approaches with the possible additional application of evolutionary algorithms, and (iv) statistical analysis, quality assessment, and functional connectivity.

**Figure 7:**
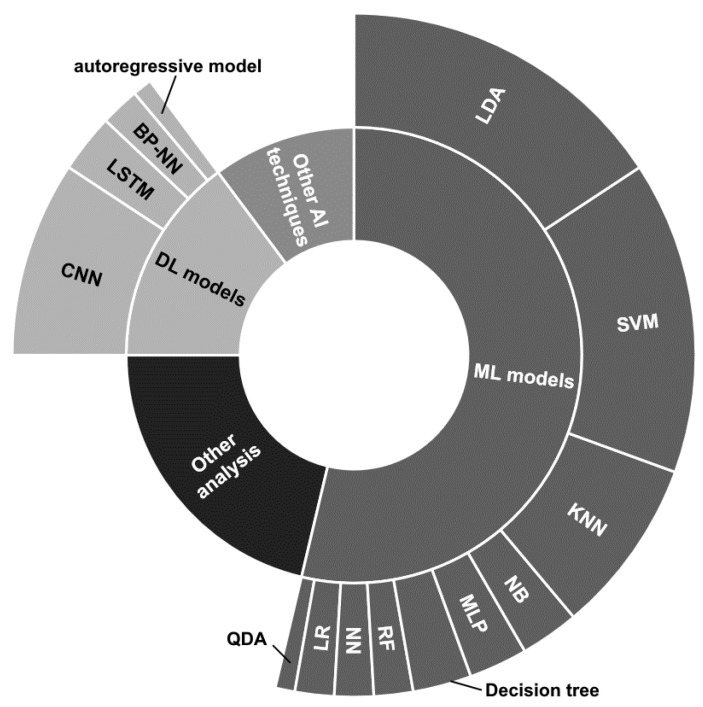
Radial graphic depicting the distribution of the classification and data analysis techniques employed by the reviewed papers.

The strategies are distributed as depicted in Figure 7. Notice that details on the proposed ML and DL models have been reported, while there are no details for the other two categories, due to the great variety of approaches.

Besides some papers that employ different strategies at the same time [89,101,115,119,121,122,134] or multiple ML techniques [89,111,133,135,144,146,152,157,183], the authors usually prefer to concentrate on a specific technique.

The traditional ML models seem to be the most used, and among them, the Linear Discriminant Analysis (LDA) classifier employed in its basic version [89,90,93,101,104,111,117,119,134,150,151,152,153,158], in combination with Common Spatial Pattern (CSP) [96], or considering multiple LDA models combined through fuzzy integration, considering optimal feature selection and classification through generalized sparse LDA [168], and presenting confidence levels assigned with particle swarm optimization [128] appears to be the most used. Likewise, the Support Vector Machine (SVM) classifier is widely employed [86,89,99,111,121,122,123,129,135,140,146,152,154,155,157,183].

The K-Nearest Neighbor (KNN) model is used by a good number of works [89,102,111,122,133,135,146,183], while the other techniques, i.e., Naive Bayes (NB) [89,111] Parzen Window [135], Multi-Layer Perceptron (MLP) [109,145,183], decision tree [89,133,183], Random Forest (RF) [157,183], neural network (NN) [111,161], Logistic Regression (LR) [100,144], and Quadratic Discriminant Analysis (QDA) [144], have been employed by a restricted number of works and together with other techniques.

Considering the DL approaches, the most used architectures have been based on the use of convolutional neural networks (CNNs) [112,121,123,126,131,142,147,163] sometimes combined with Long Short Term Memory (LSTM) layers [112,126,131]. Ref. [110] provided analyses by employing 1DCNN, 2DCCN, and one-dimensional multi-scale convolutional neural network (1DMSCNN). Instead, [143] specified the use of pre-trained CNNs, i.e., AlexNet, ResNet50, and InceptionV3. Finally, two works used a back-propagation NN [92] and an autoregressive model [120].

The *Other AI (artificial intelligence) techniques* depicted in Figure 7 refer to miscellaneous approaches that could use ensemble techniques [121], transfer learning [143] models, or other supervised learning approaches such as adaptive Riemannian classifier [130], eXtreme Gradient Boosting (XGBoost) [115], echo state network with genetic algorithm for parameter optimization and Gaussian readouts to create direction preferences [136], fuzzy integral with particle swarm optimization [134], multi-objective grey wolf optimization twin support vector machine [132], and Markov switching model [137].

Finally, a great number of statistical analysis, quality assessment, and functional connectivity studies, or other MI detection techniques, falls under the *Other analysis* label.

The applied strategies may be summarized as follows:

- A *t*-test analysis has been applied to provide alpha wave testing for comparison between systems [163], to compare average ERDs derived from different devices [162], and to compare different types of experiment [97] and experimental conditions [171];
- Questionnaire analyses have also been performed for quality assessment, by considering the opinions given by the subjects concerning a specific device [97], or employing the Quebec User Evaluation of Satisfaction with Assistive Technology test to evaluate patients’ satisfaction [88] and for subject MI ability assessment [113];
- Correlation analyses have been used to compare different electrode types [159] and to quantify subjects’ intent [115];
- MI detection through ERD only [138] or multivariate distribution [160];
- Analysis of the change in functional connectivity [103,119].

Other analyses performed by the reviewed works are the BCI-use success rate assessment based on the beta power rebound threshold [88], learning vector quantization to predict character control [124], a two-command certainty evaluation algorithm proposed by [122], the use of SPSS tool for statistic analysis [89], and the application of a transfer rate metric to determine an asynchronous real-world BCI [118].

Table 5 summarizes the information related to the classification and other analyses presented by works reporting comparisons with benchmark datasets (Section 5.4) and/or having their proprietary datasets available upon request or present in public repositories.

For each table entry, the (i) reference work, (ii) the benchmark datasets (which are detailed in Section 5.4) and their own dataset with a minimal description of the considered experimental paradigm, (iii) the classification tasks and models (if any) and other analyses (if any), (iv) the performance of the best reported model comprising the evaluation measures, the validation strategies, and the results on each of the employed datasets, and (v) if the system was tested online and/or offline, are reported.

Notice that the analyses are performed by considering the subjects one at a time, if not otherwise stated. Moreover, an asterisk (*) has been applied to all the datasets that will be detailed in the following Section 5.4, at their first appearance.

The fields of application of the reported works are very diverse; however, the classification tasks usually involve the binary classification related to the MI of hands grasping, opening/closing, or moving.

Moreover, most of the works report accuracy (usually higher than 70% for both binary and multi-class tasks) as the only performance measure to evaluate the proposed classification models, and the validation strategy is not always specified.

Finally, most of the entries present offline analyses, and just a few try to work in an online modality.

### 5.4. Dataset and Experimental Paradigms

Of the 84 cited papers, 7 report that their proprietary datasets are available upon request [93,111,119,123,133,146,174], and only [113] providing the MI-OpenBCI dataset has a publicly available resource.

Some articles present links to their data, which does not seem to be available [126,134].

Instead, the publicly available third parties’ BCI Competition III dataset IIIa [133], BCI Competition III dataset IVa [168], BCI Competition IV dataset 2a [130,133,143], 2b [110], EEG Motor Movement/Imagery Dataset [115], and the EEG BCI dataset [126] have been employed by the reviewed papers as benchmarks before proprietary data testing or to directly test the proposed approaches.

Table 6 presents a summary of the publicly available datasets, which will be described in detail in the following subsections. Notice that the reported citations in the *Dataset* field refer only to the publications presenting the dataset description or required by the dataset authors. Links to the repositories, brief information on the used technologies, and the experimental paradigms are summarized in the remaining fields.

Considering the other papers, notice that the experimental paradigms usually concern the motor imagination of left/right hand/fist [86,90,93,94,95,96,99,101,110,112,115,119,120,123,128,129,130,131,134,135,137,138,141,142,143,146,147,148,149,150,151,153,154,158,161,163,172,183], both hands [99,119,120,130,142,172], dominant or single hand movements [87,102,113,160,168], finger tapping [175], shoulder flexion, extension, and abduction [132,133], the motion of upper/lower limbs [91,97,103,104,145,177], foot/feet movement [88,100,114,119,123,129,130,142,148,172], pedaling [98,140,152], tongue movement [129], game character/robot/machinery movement control [105,106,107,108,109,117,118,119,121,122,124,125,126,127,156,174], and generic motor intention [89,155].

Peculiar experimental conditions are presented by [144,157]. Tiwari et al. [144] propose the imagination of eight cognitive tasks, i.e., forward, backward, left, right, hungry, food, water, and sleep, with the perspective of developing an efficient assistive tool for disabled people. Angrisani et al. [157] design a complex experimental paradigm of performed and imagined soft ball squeeze, dorsiflexion of the ankle, flex-extension of the forearm, finger mobilization by clenching a clothespin, and flex-extension of the leg, to validate their BCI instrumentation.

Besides the experimental paradigms considered by the reviewed works, it is interesting to have an overview of the subjects involved in the experiments.

**Figure 8:**
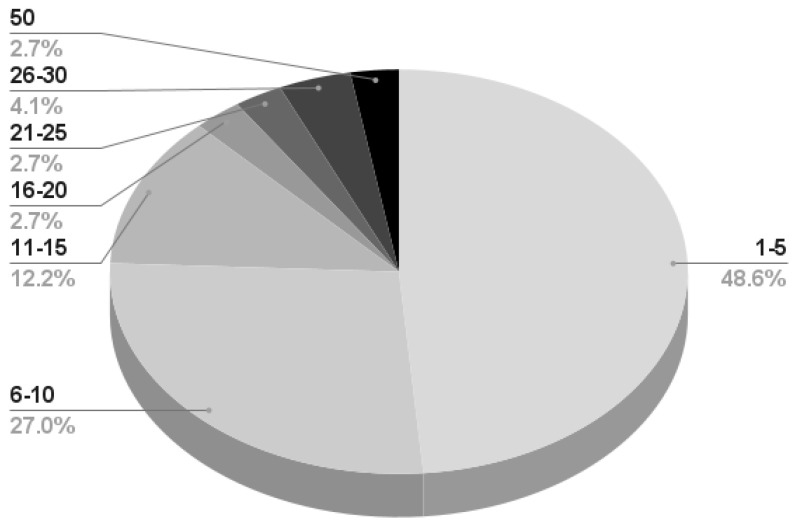
Number of subjects recruited by the remaining 79 works presenting information on the matter. The first number (bold black) represents the subject number range, while the second number (bold gray) is the percentage considering the total number of works. For example, 48% of the works presenting subject information recruit a maximum of five participants.

Excluding [115,134], which employ third parties’ datasets, framework proposals [87,106], and simulated environments [105], in the remaining 79, analysis on the involved subjects can be synthetically listed as follows:

- A total of 7/79 papers do not provide any information regarding the involved subjects;
- A total of 36/79 papers specify the biological gender of the subjects and report most of the time the number of subjects divided per male and female;
- A total of 50/79 papers recruited healthy subjects, while only 5 considered patients affected by specific pathologies;
- A total of 21/79 papers present information regarding the previous experience of the subjects with EEG, BCI, or MI-based experiments;
- A total of 35/79 papers report no information on the participants’ age, while the other works consider subjects aged around 20–30 years. Only [88,93,112] ask for the participation of adults over 30 years to a maximum of 60 years of age;
- Almost 50% of the works reporting information on the subjects perform their experiment on a maximum of 5 participants; 27% recruit a maximum of 10 subjects, and very few works consider more than 20 participants. A detailed infographic is depicted in Figure 8.

Finally, notice that of the 84 papers, 39 present an ethical statement regarding the approval of the proposed experiment, and 33 confirm that written or oral informed consent was given by the subjects.

#### 5.4.1. BCI Competition III Dataset IIIa

The BCI Competition III dataset IIIa [196] collects the data recorded from three subjects while performing a cue-based experiment of MI tasks, i.e., left/right hand, foot, or tongue movement randomly presented in six runs of 40 trials each.

The EEG signals have been acquired on 60 electrodes (the montage is depicted in the official dataset description available at https://www.bbci.de/competition/iii/desc_IIIa.pdf accessed on 31 January 2023) through a wired 64-channel Neuroscan device (250 Hz sampling rate). The reference and ground electrodes were placed on the left and right mastoids, respectively.

The output signal has been bandpass filtered (1–50 Hz), and the notch filter was enabled.

#### 5.4.2. BCI Competition III Dataset IVa

The BCI Competition III dataset IVa [196] presents the recording of five subjects, who were asked to perform left/right hand and right foot MI according to two types of visual stimulations. Each subject had to respond to 280 cues.

BrainAmp amplifiers and 128 channel Ag/AgCl electrode cap from ECI were employed for the EEG signal collection. Notice that of the 128 channels, 118 were measured at positions compliant with the extended international 10-20 system (more details on the official dataset description available at https://www.bbci.de/competition/iii/desc_IVa.html accessed on 31 January 2023).

The acquired signals were bandpass (0.05–200 Hz) filtered and digitized at 1000 Hz with 16-bit (0.1 uV) accuracy. A data version presenting downsampled signals at 100 Hz was also provided.

#### 5.4.3. BCI Competition IV Dataset 2a

The widely known BCI Competition IV dataset 2a [197] contains continuous multi-class motor imagery data acquired from nine subjects. The participants were asked to perform a cue-based MI-BCI considering the imagination of the left/right hand, both feet, and tongue movements. The subjects had to participate in two experimental sessions (six runs of 48 trials each) on different days.

Notice that the signals were recorded from 22 Ag/AgCl wired electrodes (please, consult the original publication for the montage details). Left and right mastoids presented the ground and reference electrodes, while two EOG channels were positioned to allow artifact removal. The signal presented a sampling rate of 250 Hz and was bandpass (0.5–100 Hz) and notch (50 Hz) filtered. Moreover, experts performed a manual screening of the signals and marked the trials containing artifacts.

#### 5.4.4. BCI Competition IV Dataset 2b

The *Session-to-Session Transfer of a Motor Imagery BCI under Presence of Eye Artifacts* dataset, widely known as the BCI Competition IV dataset 2b [197], was intended to provide the classification of EEG signals in the presence of ocular artifacts. Therefore, it collects EEG (on C{3,4,z} electrodes) and electrooculogram signals previously acquired by [200]. Nine right-handed healthy subjects had to perform an experiment during which they were guided to produce specific ocular artifacts, and this also presented a cue-based MI-BCI paradigm consisting of the motor imagination of left- and right-hand movements. Two sessions (each of six runs with 10 trials per run) without feedback were recorded separately for each subject. Afterward, three sessions with online feedback were performed, given that each session was constituted by four runs of 40 trials each. The feedback was in the form of a smiley changing expression and color depending on the good outcome of the MI task.

#### 5.4.5. EEG Motor Movement/Imagery Dataset

The EEG Motor Movement/Imagery Dataset available on the PhysioNet repository (https://physionet.org/content/eegmmidb/1.0.0/ accessed on 31 January 2023) [42,198] presents data acquired using a BCI2000 system and considering 64 EEG channels positioned according to the 10-10 International System (excluding electrodes Nz, F9, F10, FT9, FT10, A1, A2, TP9, TP10, P9, and P10). The recording was performed with a sampling rate of 160 Hz.

Each of the 109 subjects undertook a cue-based experiment of 14 runs consisting of two baseline recordings and three recordings per experimental task, i.e., MI and executed opening/closing of left/right fist, MI and executed opening/closing of both fists/feet.

#### 5.4.6. MI-OpenBCI

The MI-OpenBCI dataset [113] presents the recordings acquired through a consumer-grade MI-BCI system based on OpenBCI Cyton and Daisy Module. The EEG signal was recorded by using the Electrocap System II. Moreover, an electromyographic signal was acquired through the OpenBCI Ganglion board connected to the Myoware sensors. OpenViBE and OpenBCI GUI were used for EEG and electromyographic data recording, respectively.

The experiment was approved by the *Comité Asesor de Ética y Seguridad en el Trabajo Experimental* and performed by 12 (four female) healthy right-handed subjects (mean age ± SD = 25.9 ± 3.7 years) who did not have any previous experiences with BCIs. The subjects gave their informed consent. Regarding the sole EEG wireless data recording (125 Hz sampling rate), the F{z,3,4,7,8}, C{z,3,4}, T{3,4,5,6}, P{z,3,4} electrodes were employed. The reference and ground electrodes were placed at the left/right ear lobes.

The experimental protocol consisted of a cue-based motor imagination of the dominant hand grasping and a resting condition. The tasks were presented randomly 20 times (4 s) each for four runs. A 20 s baseline was acquired before the protocol started.

#### 5.4.7. EEG BCI Dataset

With the aim of providing a large and uniform dataset to design and evaluate processing strategies, [199] provides the EEG BCI dataset. The data were acquired after the approval of the *Ethics Committees of Toros University and Mersin University in the city of Mersin* (Turkey) and after having received the informed consent form signed by the subjects.

The data were acquired through a standard medical EEG station (EEG-1200 JE-921A EEG system, Nihon Kohden, Japan) considering 19 electrodes of the Electrocap System II, with varying sampling rates and an in-built filtering application.

The 13 healthy participants (five females and eight males, aged 20–35) were asked to perform different MI paradigms consisting of left/right hand, left/right leg, tongue, and finger movements.

## 6. Discussion

In this systematic review, 84 papers published in the last ten years have been deeply analyzed with the aim of answering the following main research question:

*Are wearable technologies mature for EEG-based MI-BCI applications in uncontrolled environments?*

However, several aspects should be considered to properly address this point, and thus, four sub-questions have been defined, as introduced in Section 1.

Important conclusions can be drawn to answer the first research sub-question,

RQ1: *Is there a significant amount of EEG-based MI-BCI studies using wearable technologies in the literature that implies a promising future development of this research field, especially in uncontrolled environments and outside the medical and clinical settings?*

by analyzing the results obtained through the extensive search initially performed considering different EEG, MI, and BCI related keyword combinations (Section 2.3) detailed in Table 2 and the final paper pool identified through the PRISMA flow (Figure 1).

In fact, according to the results reported in Table 2, the MI paradigm is particularly used in the EEG domain. About 26% of the works retrieved by considering only the EEG-based BCI keywords present MI paradigms, while only 0.71% present the use of wearable technologies for MI experiments.

Considering the timeline of the final filtered publications (Figure 2), most of the reviewed works have been published between 2019 and 2020, denoting the relatively new interest in wearable devices and a recent increase in the availability of these technologies to the EEG community.

Notice that around 20 different devices (Section 5.2) have been adopted in the applications reported by the 84 papers here analyzed, with different spatial resolutions (from 1 to 64 electrodes) and characteristics. This huge number of tools and variety of technical properties denote the increasing interest in this technology but make it difficult to qualitatively compare them.

Research directions have been clearly paved to provide new EEG-based MI-BCI wearable solutions with the aim of being employed for applications in heterogeneous and real-life environments.

One-third of the applications found in the reviewed literature are related to rehabilitation and assistive purposes, where the feedback of the systems plays a significant role in controlling external devices. Nearly 25% of the reviewed papers focus on methodological testing, presenting either new frameworks or particular signal processing and analysis techniques. Several works (15%) describe BCI applications developed in the entertainment field, while nearly 17% of contributions address the evaluation of new technical solutions and paradigm proposals.

Therefore, uncontrolled environments have been scrutinized by researchers to propose new EEG-based MI-BCIs wearable solutions.

Another interesting datum on the research production of the last ten years regards the development and study of BCI life-cycle pipelines, which concerns the second sub-question.

RQ2: *Are there common pipelines of processing that can be adopted from signal acquisition to feedback generation?*

Data acquisition, signal preprocessing, feature engineering and channel selection, data classification, and analyses, as well as feedback modalities of the 84 papers considered here were extensively analyzed in this review and synthesized in Section 5, and in particular in Table 5 and Figure 7. To summarize this analysis and answer RQ2, we observe that the first crucial point, especially using wireless technologies and wearable devices, is related to noise removal. To address this point, considering both internal and external noise sources, preprocessing algorithms can benefit from the knowledge of the frequencies of both the artifacts to be removed and the rhythms that should be preserved. However noise and signal frequencies often interfere.

Three main approaches can be identified, namely the use of blind source separation techniques, filters in the frequency domain, and spatial filters. The first approach usually presents the application of ICA, which separates a mixed signal into different components, assuming the presence of different signal sources. The second type of preprocessing relies on filters in the frequency domain, especially Butterworth filters, to select the brain rhythms of interest, and at the same time, remove noise. The last type of approach applies spatial filtering, like CAR filtering, taking into account the spatial correlation of the brain waves.

Even if a unique strategy is not adopted by all the applications, the noise removal procedures are quite similar among the considered publications.

For what concerns the feature engineering steps (which usually come after the preprocessing one), the variability among different papers is relatively low. In general, the ERD/ERS phenomenon is widely studied considering μ and β rhythms, exploiting time, frequency, and time–frequency handcrafted features. Only in recent years have deep learning methods begun to be used to automatically extract features from the raw signals.

Moreover, working with wearable devices and potentially in uncontrolled environments with low computational power, the reduction in data dimensionality is particularly important, especially considering the need for a low number of input data to be considered in a classification task. To this end, besides traditional feature reduction and feature selection strategies, a good number of works focus only on specific channels that are usually chosen among the central cortical area, which is coherent with the neuroscientific literature on MI.

The last processing step, represented by data analysis and classification, appears to be more heterogeneous with respect to the other ones, as depicted in Figure 7. In particular, regarding models used to perform different classification tasks, most of the works (about 54%) rely on traditional machine learning techniques, especially LDA and SVM; about 10% adopt ensemble techniques, transfer learning models, or other supervised learning approaches, while only 15% of them adopt deep learning strategies. It is also worth noting that 21% of the works do not face classification problems, but present statistical analysis, quality assessment, and functional connectivity studies.

From these considerations, we can conclude that there is a low variability in the initial steps of the whole BCI life cycle, while for what concerns data analysis and classification a higher variability can be identified. In particular, the adoption of deep learning models is at its early stage, and it is not outstanding with respect to traditional machine learning strategies.

A clear comparison and assessment of the efficacy of different classification models would benefit from the application of these strategies on data acquired using a similar experimental paradigm or on benchmark datasets.

This observation is strictly related to RQ3 and RQ4 sub-question answering. Starting from the third sub-question,

RQ3: *Are there consolidated experimental paradigms for wearable EEG-based MI-BCI applications?*

notice that the experimental paradigm adopted by most of the considered works (39 out of 84) concerns MI of left/right hand/fist movement. However a high number of different types of other MI paradigms are considered: single hand/both hands, foot/feet or tongue movement, shoulder flexion, extension and abduction, the motion of upper/lower limbs, pedaling, game character/robot/machinery movement control or generic motor intention and even the imagination of cognitive tasks. Moreover, single task duration, task order, administration modality, and experimental settings are also very heterogeneous.

From this variety of MI paradigms, several datasets have been collected or employed by the authors of the reviewed papers, allowing them to answer the last research sub-question:

RQ4: *Are there datasets available for the research community to properly compare classification models and data analysis?*

Considering data acquisition, 79 out of 84 works collect their own dataset, involving, in most of the cases (76%), less than 10 subjects. In particular, about 49% of these 79 works consider less than five participants. Notice that only seven proprietary datasets are available upon request. Moreover, among all the publicly available datasets reported in Table 6, which can be considered benchmarks, only one is acquired using wearable devices [113].

Among the 84 papers considered, only 9 adopted these benchmark datasets to evaluate the proposed models, of which 8 employed the third-party datasets acquired using wired systems.

Furthermore, notice that even if the same benchmark dataset is adopted, the classification tasks may vary from different types of binary classification: one-vs.-one (for instance, left versus right hand) or one-vs.-rest (for example, right-hand imagined movement versus resting state), and multiclass classification, with a range between three and five classes. Classification models and their performance, as well as other types of analysis, are reported in Table 5 only for those works (13 out of 84) that present results either on benchmark datasets or on available proprietary ones, making the proposed analysis reproducible. As a final important note, among these 13 publications, only 5 declare having performed online analysis.

Considering the answers to the provided sub-questions, the main research question concerning *the maturity of EEG-based MI-BCI applications in uncontrolled environments* can be addressed.

Having a closer look at the applications reported by the reviewed papers, it seems that most of them pertain to the medical and rehabilitation fields and are mostly employed in controlled environments. However, the EEG-based MI-BCI systems using wearable technologies in real-life scenarios seem to provide reliable assistance to their users and to be well received in case of assistive employment. They also seem promising in the case of entertainment, gaming, and other applications. The scenario of wearable devices available in the market is wide, also offering a huge variability in terms of electrodes, features, and costs. Even if several different computational models have been presented in the analyzed literature with promising results, the lack of reference experimental paradigms and of publicly and validated benchmark datasets acquired using wearable devices make the analysis of the model performance and the feasibility of real-time applications not completely accessible. It is unclear whether proposed strategies, often tested offline on wired benchmark datasets, can be effectively translated into online real-life wearable contexts.

Many concerns remain regarding the ethical aspects that permeate the use of these systems in environments managed by experts and in consumer-grade platforms. Concerning this point, note that among the 84 considered works, only 39 provide an ethical statement on the approval of the performed experiments.

## 7. Conclusions and Future Perspectives

The interest in EEG-based MI-BCI systems using wearable technologies has been rising in the last few years. Moreover, very different devices have been used in the analyzed studies for very diverse applications.

The experimental paradigms concerning MI tasks usually involve the motor imagination of left- and right-hand movements, even though new paradigms have been presented to address the specific needs of patients and researchers. Therefore, numerous datasets have been collected to face these demands. However, most of them are not publicly available, and testing is usually performed on recordings acquired through wired devices.

Surely, the typical steps of the BCI life-cycle appear to be maintained by most of the analyzed works. However, it is not quite clear if strategies applied to offline wired benchmark datasets can be translated into an online wireless environment.

An example that may clarify this point regards the pervasive use of ICA for signal preprocessing, which is usually performed in offline analyses. In fact, due to its methodological aspects, ICA requires an attentive evaluation of the outputted components and the identification of the artifactual ones. New ICA-based strategies have been recently proposed [201,202] to provide real-time usage of such methodologies. Therefore, future works should focus on the assessment of specific techniques developed for online analyses and concerning all the data processing steps.

Other concerns pertain to the beginning and end of the BCI life-cycle, i.e., how the non-stationarity of the EEG signal is handled and the responsiveness of the system.

In fact, the performance of EEG-based BCIs is heavily influenced by the variations due to the signal non-stationarity, especially during trial-to-trial and session-to-session transfers [203], and transitioning from the training to the feedback phase [116,204]. However, most of the available studies present insufficient information regarding the time between the system training phase and its real-time application. The reliability of the BCI in a real-world scenario should increase if the test phase shows good performances, even if taken after a long time from the training phase. Therefore, future works should consider these aspects to guarantee the applicability of BCIs in real-life contexts.

The reliability of these systems is also dependent on their responsiveness, which becomes particularly important in self-paced BCIs [205]. Feedback should be provided almost instantly to the users, who are usually trained to perform specific metal tasks [206].

The responsiveness concerning the classification and feedback time, as well as the users’ proficiency, should be documented in works concerning real-time BCIs.

Regarding other future research directions, two main fields can be identified. On the one hand, edge computing is fast evolving to improve data processing speed in real-time applications. For example, [207] overviews adaptive edge computing in wearable biomedical devices (in general, not only EEG ones), highlighting the pathway from wearable sensors to their application through intelligent learning. The authors state the following:

*The ultimate goal toward smart wearable sensing with edge computing capabilities relies on a bespoke platform embedding sensors, front-end circuit interface, neuromorphic processor and memristive devices*.

Furthermore, [208] investigates the possibility of addressing the drawbacks of wearable devices with edge computing.

The other frontier research field regards the application of quantum computing to BCI. Although efforts are only at the initial stage, some hybrid applications of quantum computing and BCI have been found, as reviewed by [209]. Recently, the authors in [210,211] discuss Quantum Brain Networks, a new interdisciplinary field integrating knowledge and methods from neurotechnology, artificial intelligence, and quantum computing. In [211], brain signals are detected utilizing electrodes placed on the scalp of a person who learns how to produce the required mental activity to issue instructions to rotate and measure a qubit, proposing an approach to interface the brain with quantum computers.

## Figures and Tables

**Table 1:** Search engine information summary. The authors names are reported with their initials.

Search Engine	Author	Last Consultation Date
IEEE Xplore	F.G.	22 June 2022
Mendeley	F.G.	22 June 2022
PubMed	S.C.	22 June 2022
ScienceOPEN	A.S.	21 June 2022
Semantic Scholar	A.S.	21 June 2022
Scopus	A.S.	22 June 2022
Web of Science	S.C.	22 June 2022
Google Scholar	M.C. and A.S.	20 June 2022

**Table 2:** Summary of the searches considering keyword subsets. The fields present the search engines and the main keywords related to the considered subsets.

Search Engine	EEG and BCI	EEG, BCI, and Wearable	EEG, BCI, and MI	EEG, BCI, Wearable, and MI
IEEE Xplore	3584	121	546	13
Mendeley	10,723	286	3118	60
PubMed	2777	57	877	14
ScienceOPEN	2659	22	303	4
Semantic Scholar	12,200	1879	3630	259
Scopus	10,733	670	2909	74
Web of Science	6564	180	2491	40

**Table 3:** EEG rhythms overview. The frequency ranges and the occurrence of the EEG rhythms are reported.

Rhythm	Frequency Range (Hz)	Occurrence
δ	≤4	infants, deep sleep
θ	4–8	emotional stress, drowsiness
α	8–13	relaxed awake state
μ	8–13	motor cortex functionalities
β	13–30	alert state, active thinking/attention, anxiety
γ	≥31	intensive brain activity

**Table 4:** Synthetic information on the wireless devices employed by the reviewed papers. For the Nautilus entries, it was unclear which device the authors used; thus, all the versions are reported. An asterisk (*) is present when the devices are amplifiers or data acquisition tools. Last information update, 3 October 2022.

Device	Producer	Electrodes	Price	Papers
B-Alert X-Series [164]	Advanced Brain Monitoring	up to 20/W (gel)	ask producer	[102]
BrainMaster Discovery 24E * [165]	bio-medical	24/W, D (compatible)	USD 5800.00	[114]
Cyton Biosensing Board * [166]	OpenBCI	8/W, D (compatible)	USD 999.00	[98,113,162]
eego rt [167]	ANT Neuro	8-64/W, D	ask producer	[168]
Enobio 20 [169]	Neuroelectrics	20/W, D	ask producer	[88,142]
Enobio 8 [170]	Neuroelectrics	8/W, D	ask producer	[93,97,148,162,171,172]
EPOC+ [173]	Emotiv	14/W (saline)	discontinued	[95,110,115,121,126,127,129,132,133,135,136,139,156,174,175]
EPOC Flex [176]	Emotiv	up to 32/W (gel, saline)	USD 1699.00–2099.00	[122,177]
EPOC X [178]	Emotiv	14/W (saline)	USD 849.00	[107]
g.USBamp * with(out) g.MOBIlab [179]	g.tec medical engineering	16/W, D	starting from EUR 11,900.00	[87,104,120,125]
Helmate [180]	abmedica	NA/NA	ask producer	[111,157]
Insight [181]	Emotiv	5/S	USD 499.00	[106]
MindWave Mobile 2 [182]	Neurosky	1/NA	USD 109.99	[108,183]
Muse headband 2 [184]	InteraXon	4/NA	EUR 269.99	[109,112,131,141,144]
g.Nautilus Multi-Purpose * [185]	g.tec medical engineering	8–64/W, D (compatible)	changing according to configuration, starting from EUR 4990.00	[89,90,105,125,130,145,146,151,153,155]
g.Nautilus PRO [186]	g.tec medical engineering	8–32/W, D	changing according to configuration, starting from EUR 5500.00	[89,90,105,125,130,145,146,151,153,155]
g.Nautilus RESEARCH [187]	g.tec medical engineering	8–64/W, D	changing according to configuration, starting from EUR 4990.00	[89,90,105,125,130,145,146,151,153,155]
NuAmps * [188]	NeuroScan	32/NA	ask producer	[92]
Quick-20 Dry EEG Headset [189]	Cognionics	19/D	ask producer	[100,137]
Starstim [190]	neuroelectrics	8–32/tES-EEG	ask producer	[103,140]
Synamps 2/RT * [191]	Neuroscan	64/NA	ask producer	[118]

**Table 5:** Synthetic information on works presenting classification and other analyses, while comparing their results with benchmark datasets or their own available upon request or published datasets. An asterisk (*) has been applied to all the datasets that will be detailed in Section 5.4, at their first appearance.

Reference	Dataset and Experimental Paradigm	Classification and/or Other Analyses	Performance of the Best Method	Online and/or Offline
Tang et al. [110]	Benchmark dataset: BCI Competition IV dataset 2b *. Own dataset: not available. Five subjects performed an experiment consisting of 90 repetitions of each of the MI tasks (left/right hand).	Classification task: binary (left vs. right hand). DBN, DWT-LSTM, 1DCNN, 2DCNN and one-dimensional multi-scale convolutional neural network (1DMSCNN).	Measures: average accuracy with 1DMSCNN. Validation strategy: dataset division in training and test sets according to the 4:1 ratio. BCI Competition IV dataset 2b (offline analysis): 82.61%. Own dataset (online analysis): accuracy for each subject 76.78%, 91.78%, 70.00%.	both
Guan, Zhao, and Yang [133]	Benchmark dataset: BCI Competition IV dataset 2a *, BCI Competition III dataset IIIa *. Own dataset: available upon request. Seven subjects performed imagination of shoulder flexion, extension, and abduction. The acquisition were repeated for 20 trials, each lasting for 5 s of activity plus 5–7 s rest.	Classification tasks: one-vs.-one, one-vs.-rest. 1. Subject-specific decision tree (SSDT) framework with filter geodesic minimum distance to Riemannian mean (FGMDRM). 2. Feature extraction algorithm combining semisupervised joint mutual information with general discriminate analysis (SJGDA) to reduce the dimension of vectors in the Riemannian tangent plane and classification with KNN.	Measures: average accuracy and mean kappa value. Validation strategy: k-fold cross validation. BCI Competition IV dataset 2a: - SSDT-FGMDRM 10-fold cross-validation average accuracy left vs. rest 82.00%, right vs. rest 81.28%, foot vs. rest 81.51%, tongue vs. rest 83.95%. SSDT-FGMDRM mean kappa value 0.589. - SJGDA 10-fold cross-validation mean accuracy left vs. rest 84.3%, right vs. rest 83.54%, foot vs. rest 82.11%, tongue vs. rest 85.23%. SJGDA mean kappa value 0.607. - SJGDA 10-fold cross-validation mean accuracy on left vs. right 79.41%, left vs. feet 87.14%, left vs. tongue 86.51%, right vs. feet 86.75%, right vs. tongue 87.00%, feet vs. tongue 82.04%. BCI Competition III dataset IIIa: 5-fold cross-validation mean accuracy on 82.78%. Own dataset: 5-fold cross-validation mean accuracy (rounded values taken from the provided bar graphics) flexion vs. rest 90.00%, extension vs. rest 80.00%, abduction vs. rest a bit more than 90.00% and flexion vs. extension 90.00%, flexion vs. abduction 95.00%, extension vs. abduction 90.00%.	offline
Peterson et al. [168]	Benchmark dataset: BCI competition III dataset IVa * and BCI competition IV dataset 2b. Own dataset: 11 subjects performed imagination of dominant hand grasping and a resting condition in four runs constituted by 20 trials per class.	Classification task: binary (rest vs. dominant hand grasping). Optimal feature selection and classification contemporaneously performed through generalized sparse LDA.	Measures: average accuracy (reported best). Validation strategy: 10 × 10 fold cross validation. BCI competition III: 90.94 (±1.06)%. BCI competition IV: 81.23 (±2.46)%. Own dataset: 82.26 (±2.98)%.	offline
Yang, Nguyen, and Chung [147]	Benchmark dataset: None Own dataset: available upon request. Six subjects, 10 trials of right hand grasping imagination for 5 s. The experiment was repeated for 10 runs. Notice that SSVEP tasks were also included.	Classification task: multi-class both MI and SSVEP. CNN.	Measures: best average accuracy (MI task). Own dataset: 91.73 (±1.55)%.	offline
Freer, Deligianni, and Yang [130]	Benchmark dataset: BCI competition IV dataset 2a. Own dataset: three subjects performed a paradigm without and with feedback. A total of 20 trials for each of the four conditions (left/right hand, both hands/feet) are performed per run with MI of 2–3 s.	Classification task: multi-class (4 classes). Adaptive Riemannian classifier.	Measures: accuracy. BCI Competition IV dataset 2a: lower than 50%. Own dataset: lower than 50%.	both
Barria et al. [88]	Benchmark dataset: None Own dataset: available (https://www.clinicaltrials.gov/ct2/show/NCT04995367 accessed on 31 January 2023). Five subjects, five phases: calibration, real movement, stationary therapy, MI detection with visual stimulation, and MI detection with visual and haptic stimulation. Besides the first phases, the protocol consisted of 10 s alternations of knee flection task and rest.	Classification task: None. Other analyses: - Control of ankle exoskeleton through knee flection. - Analysis of the success rate in using the BCI, based on the beta power rebound threshold. - Quebec User Evaluation of Satisfaction with Assistive Technology test to evaluate patients’ satisfaction.	None.	offline
Peterson et al. [113]	Benchmark dataset: None Own dataset *: https://github.com/vpeterson/MI-OpenBCI (accessed on 31 January 2023).	Classification task: binary (rest vs. dominant hand grasping). Generalized sparse discriminant analysis is used for both feature selection and classification. Other analyses: - The motor imagery ability of a single subject has been accessed through the KVIQ-10 questionnaire. - Analyses of temporal and frequency information.	Measures: average accuracy (extracted from bar plot). Own dataset: around 85% with Penalized Time–Frequency Band Common Spatial Pattern (PTFBCSP).	both
Shajil, Sasikala, and Arunnagiri [143]	Benchmark dataset: BCI competition IV dataset 2a. Own dataset: nine subjects performed 80 trials per MI conditions: left and right hand.	Classification task: binary. AlexNet, ResNet50, and InceptionV3 (pre-trained CNN models) plus transfer learning.	Measures: best average accuracy. BCI competition IV dataset 2a: InceptionV3 82.78 (±4.87)%. Own dataset: InceptionV3 83.79 (±3.49)%.	offline
Zhang et al. [115]	Benchmark dataset: used 10 subjects of Physionet EEG Motor Movement/Imagery Dataset *. Own dataset: seven subjects. Five conditions: eyes closed, left/right hand, both hands/feet paradigm (as for the benchmark dataset).	Classification task: multi-class on five conditions. RNN, CNN, RNN + CNN.	Measures: average accuracy. (Precision, Recall, F1, AUC and confusion matrix for both Physionet and own dataset are also provided). Validation: - Benchmark dataset divided into training (21,000 samples) and test sets (7000 samples). - Own dataset divided into training (25,920 samples) and test sets (8640 samples) for each subject. Benchmark dataset: best model RNN+CNN 95.53% average accuracy. Own dataset: best model RNN+CNN 94.27% average accuracy.	offline
Mwata et al. [126]	Benchmark dataset: EEG BCI dataset *. Own dataset: four subjects. Experimental conditions: right and left hand, and the neutral action.	Classification task: multi-class on three conditions (neutral, left/right with corresponding robot command forward, backward, and neutral). Hybrid CNN-LSTM model. Other analyses: Report different subject-combinations based-analysis.	Measures: average accuracy. Validation strategy: 10-fold cross validation. Benchmark dataset: 79.2%. Own dataset: 84.69%.	online
Apicella et al. [111]	Benchmark dataset: None. Own dataset: 17 subjects. Motor task consists of maintaining attention focused only on (i) the squeeze movement (attentive-subject trial), or (ii) a concurrent distractor task (distracted-subject trial); in both trials, the participant must perform the squeeze-ball movement (three sessions, 30 trials per session). Total epochs: 4590. Half of the epochs were collected during the attentive-subject trials and were labeled as belonging to the first class. The remaining part was acquired during the distracted-subject trials and was labeled as belonging to the second class.	Classification task: binary (MI during attention vs. MI during distraction). KNN, SVM, ANN, LDA, NB.	Measures: average accuracy (also provide precision, recall and F1 measure). Validation strategy: 10-fold cross validation. Own dataset: k-NN 92.8 (±1.6)%.	offline
Alanis and Gutiérrez [119]	Benchmark dataset: None. Own dataset: available upon request, two subjects, four conditions: left or right hand, both hands, move up and down both feet. Five runs of forty trials.	Classification tasks: binary one-vs.-rest. LDA classifier using features extracted by BCI2000. Other analyses: graph theory metrics to understand the differences in functional brain connectivity.	Best binary classification for both subjects: right hand open/close vs. rest. No classification results reported.	online
Mahmood et al. [123]	Benchmark dataset: None. Own dataset: available upon request, four subjects. Experimental conditions: eyes closed, left/right hand, pedal pressing.	Classification tasks: multiclass (4 classes). Population-based approach. SVM and CNN classifiers.	Measures: average accuracy. Validation strategy: 5-fold cross validation. CNN real-time accuracy: 89.65% and 93.22% for Ag/AgCl and FMNEs electrodes	both

**Table 6:** Summary of the publicly available datasets employed by some of the reviewed papers. In the *Dataset* field are reported only the citations directly related to the dataset publication.

Dataset	Link	Device	Experimental Paradigm
BCI Competition III dataset IIIa [196]	https://www.bbci.de/competition/iii/	Neuroscan, 64 channel EEG amplifier (wired)	cue-based left/right hand, foot, tongue MI
BCI Competition III dataset IVa [196]	https://www.bbci.de/competition/iii/	BrainAmps and 128 channel ECI cap (wired)	cue-based left/right hand, right foot MI
BCI Competition IV dataset 2a [197]	https://www.bbci.de/competition/iv/	22 electrodes (wired)	cue-based MI-BCI left/right hand, both feet, tongue MI
BCI Competition IV dataset 2b [197]	https://www.bbci.de/competition/iv/	3 electrodes (wired)	cue-based MI-BCI left/right hand MI
EEG Motor Movement/Imagery Dataset [42,198]	https://physionet.org/content/eegmmidb/1.0.0/	64 electrodes (wired)	cue-based motor execution/imagination left/right fist and both feet/fists opening/closing
MI-OpenBCI [113]	https://github.com/vpeterson/MI-OpenBCI	OpenBCI Cyton and Daisy Module, Electrocap System II, 15 electrodes (wearable)	cue-based dominant hand grasping MI
EEG BCI dataset [199]	https://figshare.com/collections/A_large_electroencephalographic_motor_imagery_dataset_for_electroencephalographic_brain_computer_interfaces/3917698	EEG-1200 JE-921A EEG system, 19 electrodes (wired)	left/right hand, left/right leg, tongue, and finger MI

All links accessed on 31 January 2023.

## Data Availability

Not applicable.

## Supplementary Materials

The following supporting information can be downloaded at https://www.mdpi.com/article/10.3390/s23052798/s1. Table S1: table reporting detailed notes on the 84 reviewed papers. The notes are organized to provide a clear reference to the papers and to follow the core section of the review (Section 5), analyzing (i) field of applications, (ii) employed technologies, (iii) signal processing and analysis methodologies, (iv) BCI feedback, and (v) dataset information.

## Abbreviations

The following abbreviations are used in this manuscript:

1DMSCNN	One-Dimensional Multi-Scale Convolutional Neural Network
AI	Artificial intelligence
ANN	Artificial neural network
AUC	Area Under the Curve
BCI	Brain–computer interface
CAR	Common Average Reference
CNN	Convolutional neural network
CSP	Common Spatial Pattern
DBN	Deep Belief Network
DL	Deep learning
ECG	Electrocardiogram
ECoG	Electrocorticography
EEG	Electroencephalography
EMG	Electromyography
EOG	Electrooculography
ERD	Event-Related Desynchronization
ERP	Event-Related Potentials
ErrP	Error-Related Potential
ERS	Event-Related Synchronization
FFT	Fast Fourier Transform
FGMDRM	Framework with filter geodesic minimum distance to Riemannian mean
FMRI	Functional Magnetic Resonance Imaging
ICA	Independent Component Analysis
KNN	K-Nearest Neighbor
LDA	Linear Discriminant Analysis
LPA	Left pre-auricolar point
LR	Logistic Regression
LSTM	Long-Short Term Memory
MI	Motor imagery
ML	Machine Learning
MLP	Multi-Layer Perceptron
MSCNN	Multi-Scale Convolutional Neural Network
NB	Naive Bayes
NN	Neural network
PRISMA	Preferred Reporting Items for Systematic Reviews and Meta-Analyses
PSD	Power Spectral Density
PTFBCSP	Penalized Time–Frequency Band Common Spatial Pattern
QDA	Quadratic Discriminant Analysis
RF	Random forest
RNN	Recurrent neural network
RPA	Right pre-auricolar point
RQ	Research question
SJGDA	Semisupervised Joint mutual information with General Discriminate Analysis
SNR	Signal to Noise Ratio
SSDT	Subject specific decision tree
SSVEP	Steady-state visual evoked potential
SVM	Support vector machine
TES	Transcranial electrical stimulation
VR	Virtual reality
XGBoost	Extreme Gradient Boosting
