# Supplementary material for: EEG-Based BCIs on Motor Imagery Paradigm Using Wearable Technologies: A Systematic Review
Source: Sensors (Basel). 2023 Mar 3;23(5):2798. doi: 10.3390/s23052798 (PMC10007053; doi:10.3390/s23052798)
Supplement: Supplementary file 1 [file sensors-23-02798-s001.zip › sensors-2225502-supplementary.pdf]

Table S1. Detailed notes on the 84 reviewed papers. The notes are organized to provide a clear reference to the papers and to follow the core section of the review (Section 5), analyzing (i) field of applications, (ii) employed technologies, (iii) signal processing and analysis methodologies, (iv) BCI feedback, and (v) dataset information.

**Reference**	**Application**	**Technologies (wireless, electrode type, storage, communication protocol)**		**Signal processing & analyses**			**Feedback**	**Dataset**
		**EEG related**	**other**	**pre-processing**	**feature engineering**	**classification and/or other analyses**		
Abdalsalam, E., Yusoff, M. Z., Malik, A., Kamel, N. S., & Mahmoud, D. (2018). Modulation of sensorimotor rhythms for brain-computer interface using motor imagery with online feedback. Signal, Image and Video Processing, 12(3), 557-564.	Impact of Motor Imagery (MI) and associated feedback on sensorimotor rhythms assessment for Brain Computer Interface (BCI) control.	Enobio 8: medically certified 8 dry/wet electrodes wearable device with flexible positioning of the electrodes, according to the 10/20 international system. Employed electrodes: F3, F4, T7, C3, Cz, C4, T8, and Pz.<br><br>Sampling rate: 500Hz.	BCI2000 system for online processing.	Online: Laplacian filtering through BCI2000.	Online: alpha and beta power estimation through bandpass filtering for Event Related Desynchronization/Synchronization (ERD/ERS) computation.	Offline analysis (on calibration data): visual inspection of EEG spectra and coefficient of determination computation.<br><br>Classification performed but strategy not specified.	Cursor movement on screen.	Not available
Abdulwahab, S. S., Khleaf, H. K., & Jassim, M. H. (2021, August). EEG Motor-Imagery BCI System Based on Maximum Overlap Discrete Wavelet Transform (MODWT) and cubic SVM. In Journal of Physics: Conference Series (Vol. 1973, No. 1, p. 012056). IOP Publishing.	Testing of different (Support Vector Machine) SVM kernels after feature extraction based on maximum overlap wavelet transform.	Emotiv EPOC+: 10/20 international system, 14 electrodes (AF3, AF4, F7, F3, F4, F8, FC5, FC6, T7, T8, P7, P8, O1, O2) plus 2 reference electrodes.	NA	Chebyshev Type II lowpass filter (40 Hz cutoff).	Maximum overlap discrete wavelet transform to extract feature concerning all the EEG rhythms.	Different SVM kernel testing led to better performances derived from the application of cubic SVM.	None	Not available
Alanis-Espinosa, M., & Gutiérrez, D. (2020). On the assessment of functional connectivity in an immersive brain-computer interface during motor imagery. Frontiers in Psychology, 11, 1301.	BCI system for the interaction with a robot through different immersion experiences. Users control the movement of a NAO humanoid robot as if the movement of the robot was his/her own.	Electrode cap: 32-channel EEG system (Mobita TMSi).	Virtual reality headset.	Bandpass filtered with a zero-phase fourth-order Butterworth (1 - 100 Hz) filter and notch filtering (60 Hz) to remove artifact caused by electrical power lines.<br><br>The blinking artifacts were removed using Indepentent Component Analysis (ICA).	The coefficient of determination (r2 value) provides a measure of how strongly the means of two distributions differ in relation to variance. r2 is calculated for each couple of electrodes considering their PSD.<br>The channels and frequencies with higher r2 values are selected as features to train the classifier.<br><br>Partial directed coherence is used to measure the coupling or connectivity between different channels in the frequency domain.	Actions: open/ close left or right hand, left and right hands, move up and down both feet.<br>Binary classifiers: each of the tested classes compared against the resting state.<br><br>Graph theory metrics to understand the differences in functional brain connectivity.<br><br>Linear Discriminant Analysis (LDA) classifier is used to discriminate between the control command (detection of features that characterize the MI task) or the rest state.	Feedback from the robot.	Available upon request
Angrisani, L., Arpaia, P., Donnarumma, F., Esposito, A., Frosolone, M., Improta, G., ... & Parvis, M. (2020, May). Instrumentation for Motor Imagery-based Brain Computer Interfaces relying on dry electrodes: a functional analysis. In 2020 IEEE International Instrumentation and Measurement Technology Conference (I2MTC) (pp. 1-6). IEEE.	Presentation of new instrumentation and functional analysis of a wireless wearable helmet with 8 dry electrodes.	Helmet (ab medica Helmate): 8 channels (Fp1, Fp2, Fz, Cz, C3, C4, O1, O2) and 10 dry electrodes.	Helmet communicates via Bluetooth with a PC.	Bandpass (1 - 45 Hz) and notch (50 Hz) filtered.	Common Spatial Pattern (CSP).	Five exercises have been executed: squeeze a soft ball, dorsiflexion of the ankle, flex-extension of the forearm, finger mobilization by clenching a clothespin, and flex-extension of the leg. Therefore, five binary classifications comparing each exercise with respect to left and right imagined movement are performed.<br><br>Classification performed by using Random Forest (RF) and SVM with Gaussian kernel.	None	Not available
Apicella, A., Arpaia, P., Frosolone, M., & Moccaldi, N. (2021). High-wearable EEG-based distraction detection in motor rehabilitation. Scientific Reports, 11(1), 1-9.	EEG-based distraction detection: during a rehabilitation motor task, EEG trend is influenced by the state of the patient attention or distraction to the task itself.	AB-Medica Helmate 8, dry electrodes, 8 channels.	Use dedicated software (Helm8 Software Manager).	Acquisition software allows to use several filters (e.g., notch and IIR). Artifact removal (ICA).	Relative and absolute PSD.<br><br>Features (frequency domain): 7 traditional EEG bands, nine 8 Hz bands, 12 4 Hz bands.<br>Features (time domain): CSP preceded or not by different types of Filter-Banks.	Classifiers: kNN (k-Nearest Neighbor), SVM, ANN (Artificial Neural Network), LDA, NB (Naive Bayes).<br><br>Best accuracy achieved with k-NN (92.8 ± 1.6%).	None	Available upon request
Arfaras, G., Athanasiou, A., Pandria, N., Kavazidi, K. R., Kartsidis, P., Astaras, A., & Bamidis, P. D. (2017, June). Visual versus kinesthetic motor imagery for BCI control of robotic arms (Mercury 2.0). In 2017 IEEE 30th International Symposium on Computer-Based Medical Systems (CBMS) (pp. 440-445). IEEE.	Control of house developed robotic arms (8 degrees of freedom).<br>Compare two different modalities of MI: visual observation (VMI) and kinesthetic rehearsal (KMI) of movements.	Emotiv Epoc.	Emotiv Cognitiv suite to train the BCI (for each subject).	Not specified (through Emotiv Cognitiv Suite).	Not specified (through Emotiv Cognitiv Suite).	Not specified (through Emotiv Cognitiv Suite).	None	Not available

Table S1. Detailed notes on the 84 reviewed papers. The notes are organized to provide a clear reference to the papers and to follow the core section of the review (Section 5), analyzing (i) field of applications, (ii) employed technologies, (iii) signal processing and analysis methodologies, (iv) BCI feedback, and (v) dataset information.

**Reference**	**Application**	**Technologies (wireless, electrode type, storage, communication protocol)**		**Signal processing & analyses**			**Feedback**	**Dataset**
		**EEG related**	**other**	**pre-processing**	**feature engineering**	**classification and/or other analyses**		
Athanasiou, A., Arfaras, G., Xygonakis, I., Kartsidis, P., Pandria, N., Kavazidi, K. R., ... & Bamidis, P. D. (2017, June). Commercial BCI Control and functional brain networks in spinal cord injury: a proof-of-concept. In 2017 IEEE 30th International Symposium on Computer-Based Medical Systems (CBMS) (pp. 262-267). IEEE.	Control of a robotic arm for movement restoration and rehabilitation of spinal cord Injury patients.	Use of both wired and wireless EEG:<br>- Nihon-Kohden (Japan) 128-channel EEG with a Brain Products active electrodes cap using the 10/5 international electrode system.<br>- Emotiv Epoc + with 14 channels.	NA	Signal pre-processing through MATLAB:<br>- band-pass (Finite Impulse-Response) FIR filter (0.5 - 30 Hz)<br>- EEGLAB toolbox to perform INFOMAX ICA.	Not specified.	Classification of rest, left/right hand conditions and functional connectivity analyses.	Robotic arm movement.	Not available
Barria, P., Pino, A., Tovar, N., Gomez-Vargas, D., Baleta, K., Díaz, C. A., ... & Cifuentes, C. A. (2021). BCI-Based Control for Ankle Exoskeleton T-FLEX: Comparison of Visual and Haptic Stimuli with Stroke Survivors. Sensors, 21(19), 6431.	Control of ankle exoskeleton through brain computer interfacing.	Enobio 20, 500 Hz frequency rate, and high dynamic resolution (24 bits, 0.05 uV).<br>Employed electrodes: C1, C2, FCz, and CPz. Cz used as the reference electrode.<br><br>NIC 2.0 Software for motor cortex recording.<br><br>OpenVibe to process EEG data in real-time.	T-FLEX ankle exoskeleton.<br><br>A stimulation was given to help performing the motor imagery task.	Laplacian spatial filter and a 4th-order Butterworth bandpass filter (pass band ripple of 0.5 dB) with cutoff between 16 and 24 Hz.	Consider threshold on beta power rebound.<br><br>PSD to estimate the variation of energy present in the Event Related Potential (ERP).	Analysis on the success rate in using the BCI, based on the beta power rebound threshold.<br><br>Quebec User Evaluation of Satisfaction with Assistive Technology test to evaluate patients' satisfaction.	T-FLEX movement.	See: https://www.clinicaltrials.gov/ct2/show/NCT04995367<br>.
Bista, S., & BikramAdhikari, N. (2018, October). Performance Analysis of Tri-channel Active Electrode EEG Device Designed for Classification of Motor Imagery Brainwaves for Brain ComputerInterface. In 2018 International Conference on Advances in Computing, Communication Control and Networking (ICACCCN) (pp. 662-667). IEEE.	Design a simple, low cost, 3-channel active electrode EEG data acquisition device.	Self-made EEG device: C3, Cz, and C4 electrodes.	The data acquisition tool consists of an Arduino-Uno hardware and MATLAB Software communicating via USB connection.	Notch filter (50Hz) followed by ICA to remove ocular and muscular artifacts.	Wavelet decomposition.	LDA for binary classification: right/left hand.	None	Not available
Cardoso, V. F., Delisle-Rodriguez, D., Romero-Laiseca, M. A., Loterio, F. A., Gurve, D., Floriano, A., ... & Freire Bastos-Filho, T. (2021). Effect of a Brain–Computer Interface Based on Pedaling Motor Imagery on Cortical Excitability and Connectivity. Sensors, 21(6), 2020.	Cycling based BCI.	Open BCI Cyton board, 8 EEG channels (FC1, FC2, C3, C4, Cz, CP1, CP2, and Pz), 250 Hz sampling rate.	BCI also composed of a notebook, a Raspberry Pi board, and a motorized pedal.	Notch filter (60 Hz).	Analysis of ERD patterns into the time–frequency representation, relative power into the frequency domain, and EEG connectivity.	Not specified.	Passive pedaling feedback.	Not available
Carrino, F., Dumoulin, J., Mugellini, E., Abou Khaled, O., & Ingold, R. (2012, January). A self-paced BCI system to control an electric wheelchair: Evaluation of a commercial, low-cost EEG device. In 2012 ISSNIP biosignals and biorobotics conference: biosignals and robotics for better and safer living (BRC) (pp. 1-6). IEEE.	GERBIL (self-paced BCI) application for wearable EEG signal processing.<br><br>Based on OpenViBE.	Emotiv Epoc chosen after considering other two devices OCZ NIA and Neurosky MindSet.<br><br>Experiment: MindMedia NeXuS 32b. Channels: C3;C4;FC3;FC4;C5;C1;C2;C6;CP3;CP4.	Bluetooth for communication.<br><br>Virtual Reality Peripheral Network (based on TCP/IP protocol) to guarantee the communication with the GERBIL application.	Laplacian surface filtering (through OpenViBE). Provide two signals corresponding to the sensorimotor cortex related to left/right hand movement.<br><br>Temporal filter to extract the bandwidth (8 - 24 Hz).<br><br>Temporal epoching.	Feature extracted through OpenViBE.	LDA (k-fold cross validation) for classification of left/right hand movement.<br><br>GERBIL provides the command to the wheelchair by checking the classification ouput continuously.	Command a smart wheelchair.	Not available
Cha, K., Lee, J., Kim, H., Kim, C., & Lee, S. (2019, February). Steady-State Somatosensory Evoked Potential based Brain-Computer Interface for Sit-to-Stand Movement Intention. In 2019 7th International Winter Conference on Brain-Computer Interface (BCI) (pp. 1-3). IEEE.	Simultaneous hybrid BCI by combining MI with vibrotactile stimulation.	g.Nautilus: 32-channel wireless EEG system.	Custom made vibrotactile motors.	Band-pass filtered (0.5 - 100 Hz). Notch filtered (60 Hz).	Feature extraction with Filter Bank CSP and feature selection with MIBIF.	SVM to detect sit to stand movement intention.	None	Not available

Table S1. Detailed notes on the 84 reviewed papers. The notes are organized to provide a clear reference to the papers and to follow the core section of the review (Section 5), analyzing (i) field of applications, (ii) employed technologies, (iii) signal processing and analysis methodologies, (iv) BCI feedback, and (v) dataset information.

**Reference**	**Application**	**Technologies (wireless, electrode type, storage, communication protocol)**		**Signal processing & analyses**			**Feedback**	**Dataset**
		**EEG related**	**other**	**pre-processing**	**feature engineering**	**classification and/or other analyses**		
Daeglau, M., Wallhoff, F., Debener, S., Condro, I. S., Kranczioch, C., & Zich, C. (2020). Challenge accepted? Individual performance gains for motor imagery practice with humanoid robotic EEG neurofeedback. Sensors, 20(6), 1620.	Neurofeedback through foot MI practice (preceded by real movements) with humanoid robot.<br><br>The paradigm consisted in a race between a brain commanded robot and a computer commanded one, after a training phase divided in 2 blocks, to investigate social effects on neurofeedback.	SMARTING: 24 electrodes, with FCz as ground and Afz as reference electrodes.<br><br>Sampling rate: 500 Hz.	NAO humanoid robot.<br><br>Data acquisition through OpenVibe.	Online analysis<br>- High pass (8 Hz) and low pass (30 Hz) FIR filtering (EEGLAB).<br>- Signal epoching.<br>Offline analysis:<br>- High pass (1 Hz) and low pass (40 Hz) FIR filtering (EEGLAB).<br>- Signal epoching plus epoch rejection when presenting artifacts. Use of ICA to discard specific epochs.<br>- After artifact rejection, the signal was low pass (30 Hz) and high pass (8 Hz) FIR filtered. Improbable channels were identified through an EEGLAB extension function. Signal were further segmented and outliers removed.	Online feature: ERD.<br>Offline features: NA.<br><br>Online analysis: Selection of a single channel with a stronger relative ERD depending on the data of 2 executed experiment blocks.<br>Offline analysis: choose a region of interest comprising channels Cz, CP1, CPz, and CP2.	Online analysis: LDA classification plus relative ERD.<br><br>Offline analysis: statistical analysis.	Robot movement.	Not available
Dehzangi, O., Zou, Y., & Jafari, R. (2013, November). Simultaneous classification of motor imagery and SSVEP EEG signals. In 2013 6th International IEEE/EMBS Conference on Neural Engineering (NER) (pp. 1303-1306). IEEE.	Simultaneous classification of MI and steady-state visual evoked potentials (SSVEP) tasks.	Data acquisition system, designed and developed by the authors: set of active dry-contact electrodes, 8 channels (C3, C4, FC3, FC4, P07, P08, Oz, and POz).	The system also consists of alow-noise recording electronics including Texas Instruments ADS1299 and<br>Bluetooth low energy communication module.	Filtered (0.5 - 30 Hz).	C3, C4, FC3, FC4 electrodes used for MI and the rest for SSVEP task.<br><br>Extracted features: band power, fractal dimension and wavelet packet tree.	SVM classifier to distinguish the imaginary right and left hand movements.	None	Not available
Djamal, E. C., Abdullah, M. Y., & Renaldi, F. (2017). Brain computer interface game controlling using fast fourier transform and learning vector quantization. Journal of Telecommunication, Electronic and Computer Engineering (JTEC), 9(2-5), 71-74.	BCI for game controlling.	Neurosky with Fp1 channel.	NA	Use Fast Fourier Transform (FFT) to move in the frequency domain.	Not specified.	Learning vector quantization to predict upward or downward movement for character control.	Movement of the game character.	Not available
Du Bois, N., Bigirimana, A. D., Korik, A., Kéthina, L. G., Rutembesa, E., Mutabaruka, J., ... & Coyle, D. H. (2021). Neurofeedback with low-cost, wearable electroencephalography (EEG) reduces symptoms in chronic Post-Traumatic Stress Disorder. Journal of affective disorders, 295, 1319-1334.	Effect of NeuroFeedback training on patients with syntoms of post-traumatic stress disorder.	High resolution EEG for one participant: Nautilus Pro and Nautilus Ladybird, 32 channels. FlexEEG 8-channel passive electrode EEG headset (NeuroCONCISE, 2021) for rest of participants.	Use NeuroroSensi games platform.	Frequency bands filtering: 8-12Hz (alpha), 12-18Hz (low beta), 18-28Hz (high beta), and 28-40Hz (low gamma).<br><br>High-pass and low-pass FIR filter modules (band-pass attenuation 0dB, band-stop attenuation 60 dB).	CSP and mutual information.	LDA classifier for binary classification of left or right hand MI.	Feedback was presented visually via a videogame.	Not available
Freer, D., & Yang, G. Z. (2020). MIndGrasp: A New Training and Testing Framework for Motor Imagery Based 3-Dimensional Assistive Robotic Control. arXiv preprint arXiv:2003.00369.	Assistive robotic control. Train a BCI in a simulated environment that resembles real-world assistive scenarios.	g.Nautilus: 32-channel.	Robotic arm	Not specified	Not specified	Classifier: Riemannian MDM.<br>4 classes: left, right, both hands, both feet.	Grasping objects.	SIMULATED EEG
Freer, D., Deligianni, F., & Yang, G. Z. (2019, May). Adaptive Riemannian BCI for enhanced motor imagery training protocols. In 2019 IEEE 16th International Conference on Wearable and Implantable Body Sensor Networks (BSN) (pp. 1-4). IEEE.	Improve real-time feedback through adaptive Riemannian BCI.	g.Nautilus: 32-channel wet.	NA	Not specified	Not specified	Adaptive Riemannian classifier to detect 4 different MI conditions (left/right hand, both hands/feet).	None	BCI competition IV dataset 2a and own dataset (not available).
Gant, K., Guerra, S., Zimmerman, L., Parks, B. A., Prins, N. W., & Prasad, A. (2018). EEG-controlled functional electrical stimulation for hand opening and closing in chronic complete cervical spinal cord injury. Biomedical physics & engineering express, 4(6), 065005.	BCI-FES system for spinal cord injury patients restoration. The authors propose an open and closed loop BCI-FES configuration.	20-channel (used Cz, C1, C2, C3, C4) wireless EEG system (B-Alert X24, Advanced Brain Monitoring, Carlsbad, CA). Reference electrodes placed on mastoids.	Bluetooth for communication.<br><br>Functional Electrical Stimulation (FES) devices.	4th order bandpass filter (5 - 35 Hz).	Not specified	Online decoding with SVM classifier.<br><br>Wilcoxon rank sum test to determine whether there were significant differences in accuracies between spinal cord injured subjects and control ones.	FES and hand opening/closing if intention correctly classified.	Not available

Table S1. Detailed notes on the 84 reviewed papers. The notes are organized to provide a clear reference to the papers and to follow the core section of the review (Section 5), analyzing (i) field of applications, (ii) employed technologies, (iii) signal processing and analysis methodologies, (iv) BCI feedback, and (v) dataset information.

Reference	Application	Technologies (wireless, electrode type, storage, communication protocol)		Signal processing & analyses			Feedback	Dataset
		EEG related	other	pre-processing	feature engineering	classification and/or other analyses		
Garcia-Moreno, F. M., Bermudez-Edo, M., Garrido, J. L., & Rodríguez-Fórtiz, M. J. (2020). Reducing response time in motor imagery using a headband and deep learning. Sensors, 20(23), 6730.	Investigate the smallest response time that obtains a competitive accuracy on the detection of MI using deep learning.	Muse headband version 2 by InteraXon considering 4 channels: AF7, AF8 TP9, TP10.	Mind Monitor application to record the brain signals in real-time.	Notch filter (50Hz) and noise removal using Exploratory Data Analysis (EDA).	Concatenation of ♂alpha, beta, gamma, theta and delta waves + deep features extracted from 1D-CNN (Convolutional Neural Network) layer.	Deep learning architecture based on 1D-CNN and Long Short Term Memory (LSTM) layers. Classification left/right hand.	None	Not available
Garcia-Moreno, F. M., Bermudez-Edo, M., Rodríguez-Fórtiz, M. J., & Garrido, J. L. (2020, July). A CNN-LSTM deep Learning classifier for motor imagery EEG detection using a low-invasive and low-Cost BCI headband. In 2020 16th International Conference on Intelligent Environments (IE) (pp. 84-91). IEEE.	Detect EEG-MI (left and right hand) as a first assessment prior to assessment of user's (especially elderly's) independence in using low-cost and non-invasive devices.	Muse headband 2: 10/20 international system, 4 goldplated cup bipolar electrodes (TP9, AF7, AF8, TP10). The Fpz electrode is used only as a reference.<br><br>Mind Monitor mobile application for real-time signal recording.<br><br>Sampling rate: 250Hz.	Bluetooth pairing between Muse headband and mobile phone.	Mind Monitor direct rhythm (delta, theta, alpha, beta, gamma) extraction through Power Spectral Density (PSD).<br><br>Different window sizes tested with 50% overlap.	Using directly a CNN layer in the proposed DL model for feature extraction.	3 users for model evaluation (90% of each user data for training and 10% for validation) and 1 for testing.<br><br>DL model (Python and Keras library):<br>- 1 Input layer 3D (sample, timesteps, features).<br>- 1 CNN layer (32 filters of size 1) to extract relevant features from the brain waves corresponding to the 5 rhythms.<br>- 1 LSTM layer (32 neurons, 0.2 dropout, 0.001 regularizer) to classify the time series.<br><br>Perform parameter tuning and model evaluation with accuracy and MSE.	None	Not available
Gaxiola-Tirado, J. A., Iáñez, E., Ortíz, M., Gutiérrez, D., & Azorín, J. M. (2019, July). Effects of an exoskeleton-assisted gait motor imagery training in functional brain connectivity. In 2019 41st Annual International Conference of the IEEE Engineering in Medicine and Biology Society (EMBC) (pp. 429-432). IEEE.	Robotic exoskeleton for gait rehabilitation.	StarStim R32 system (Neuroelectrics). EEG signals from 30 channels placed on the scalp<br>according to the extended 10/20 system (P7, P4, CZ, PZ, P3, P8, O1, O2, C2, C4, F4, FP2, FZ, C3, F3, FP1, C1, OZ, PO4, FC6, FC2, AF4, CP6, CP2, CP1, CP5, FC1, FC5, AF3, PO3)<br>at a sampling frequency of 500 Hz.	Communication between exoskeleton and the computer through a bluetooth port using MATLAB.	Band-pass filter (0.5 - 50 Hz), notch filter (50 Hz) and Laplacian filter.<br>ICA analysis with EEGLAB toolbox to detect visually the presence of blinking artifacts.	Partial directed coherence analysis.	Analysis of the change of functional connectivity.	Exoskeleton movement.	Not available
Guan, S., Li, J., Wang, F., Yuan, Z., Kang, X., & Lu, B. (2021). Discriminating three motor imagery states of the same joint for brain-computer interface. PeerJ, 9, e12027.	Proposal of a new BCI framework including feature extraction and classification for motor imagery.	Emotiv Epoc + with 14 channels (AF3, F7, F3, FC5, T7, P7, O1, O2, P8, T8, FC6, F4, F8, and AF4) and 2 reference electrodes (CMS, DRL).	NA	5th order Butterworth filter (8 - 30 Hz); automatic artifact removal toolbox to remove ocular and muscular artifacts; Common Average Reference (CAR) to reduce background noise; endpoint effect deletion through mirror extending technology.	Based on local mean decomposition (LMD), cloud model, and CSP, a feature extraction method called LMD-CSP is proposed to extract distinguishable features.	Multi-objective grey wolf optimization twin support vector machine to classify shoulder abduction, extension, and flexion MI.	None	Not available
Guan, S., Zhao, K., & Yang, S. (2019). Motor imagery EEG classification based on decision tree framework and Riemannian geometry. Computational intelligence and neuroscience, 2019.	Methodological for multi-class MI problem classification (shoulder flexion, extension,<br>and abduction).	Emotiv EPOC+: 10/20 international system, 14 electrodes (AF3, AF4, F7, F3, F4, F8, FC5, FC6, T7, T8, P7, P8, O1, O2) plus 2 reference electrodes.<br><br>Sampling rate: 128Hz.	NA	Not specified	Feature extraction obtained by combination of semisupervised joint mutual information with general discriminate analysis (SJGDA) to reduce the dimension of vectors in the Riemannian tangent plane.	1. Subject-specific decision tree framework with filter geodesic minimum distance to Riemannian mean for multi-class problem.<br>2. SJGDA and subject-specific decision tree kNN for multi-class problem after tangent space mapping.<br><br>Tested on 3 dataset: BCI Competition IV dataset 2a, BCI Competition III dataset IIIa, own dataset.	None	The BCI Competition datasets are available online, while the authors' dataset is available upon request.
Hirsch, G., Dirodi, M., Xu, R., Reitner, P., & Guger, C. (2020, July). Online classification of motor imagery using EEG and fNIRS: a hybrid approach with real time human-computer interaction. In International Conference on Human-Computer Interaction (pp. 231-238). Springer, Cham.	Real- time hybrid BCI for MI, combining EEG and fNIRS.	Wireless g.Nautilus fNIRS (g.tec) with 16 channels of EEG, combined with 8 channels of fNIRS.	NA	Band pass filtered with a 6th-order Butterworth filter (8 - 30 Hz) to limit the bandwidth to the alpha/mu and beta band. 2nd-order notch filter (50 Hz) to remove power line interference.	CSP	LDA detect right/left grasp.	Direct positive feedback is provided for the subjects to facilitate the imagination of the movements.	Not available

Table S1. Detailed notes on the 84 reviewed papers. The notes are organized to provide a clear reference to the papers and to follow the core section of the review (Section 5), analyzing (i) field of applications, (ii) employed technologies, (iii) signal processing and analysis methodologies, (iv) BCI feedback, and (v) dataset information.

Reference	Application	Technologies (wireless, electrode type, storage, communication protocol)		Signal processing & analyses			Feedback	Dataset
		EEG related	other	pre-processing	feature engineering	classification and/or other analyses		
Jameel, H. F., Mohammed, S. L., & Gharghan, S. K. (2019, October). Electroencephalograph-based wheelchair controlling system for the people with motor disability using advanced brainwear. In 2019 12th International Conference on Developments in eSystems Engineering (DeSE) (pp. 843-848). IEEE.	EEG wheelchair control system for disabled people.	Emotiv Insight (using electrodes AF3, AF4, T7, T8, Pz).	DC motor driver, microcontroller, DC motor, microwave sensor, arduino for electric wheelchair.	not clearly specified	not clearly specified	not clearly specified	Wheelchair movement.	Not available
Jawanjalkar, A. R., & Padole, D. V. (2017, March). Development of soft computing technique for classification of EEG signal. In 2017 International Conference on Innovations in Information, Embedded and Communication Systems (ICIIECS) (pp. 1-6). IEEE.	Soft computing techniques for EEG data classification.	The acquisition device consists of a pre-amplifier unit, a microcontroller unit,<br>and a Bluetooth transmit unit.<br><br>The EEG device uses dry electrodes.	NA	Noise removal (not specified).	Sub-band CSP.	LDA and fuzzy integral with Particle Swarm Optimization (PSO).	None	The authors report the following link, which is not available:http://www.bsp.brain.riken.jp/~qibin/homepage/Datasets.html
Jiang, Y., Hau, N. T., & Chung, W. Y. (2017). Semiasynchronous BCI using wearable two-channel EEG. IEEE Transactions on Cognitive and Developmental Systems, 10(3), 681-686.	Propose a semiasynchronous MI-based BCI applying discrete and continuous feedback.	Proposed wearable wireless EEG system: headset (2 channels C3 and C4) + PC based<br>signal processing module. EEG headset is comprised of a sensory detection module and a sensory processing module.	The digitized data is wirelessly transmitted to the PC via a Bluetooth low-energy<br>module.	Not specified	To extract features, the ERD/ERS in alpha band (8 - 12 Hz) was measured.	SVM to detect left/right hand MI.	Discrete feedback for training and continuous feedback for testing.	Not available
Karakullukcu, N., & Yilmaz, B. (2022). Detection of Movement Intention in EEG-Based Brain-Computer Interfaces Using Fourier-Based Synchrosqueezing Transform. International Journal of Neural Systems, 32(01), 2150059.	Identify and characterize movement intention (resting and MI) using multichannel EEG signals and Fourier-based synchrosqueezing transform, to assist people affected by motor impairments.	Active dry g.Sahara cap (Cz, FP2, F3, Fz, F4, T7, C3, FP1, C4, T8, P3, Pz, P4, PO7, PO8 and Oz) and g.Nautilus amplifier.<br>Reference electrodes were placed behind the ear lobes.<br><br>Sampling rate: 500 Hz.	NA	In hardware bandpass (2 - 30 Hz) and notch (50 Hz) filtering.<br><br>The authors perform manual pre-processing of the signal through MATLAB.<br><br>FP1 and FP2 channels removed due to highly corrupted signal by muscle movement.<br>The authors selected manually the trials with less noise.	In order to determine the best features, 12 methods were used: statistical features (e.g., skewness, log energy entropy, Shannon entropy, kurtosis and energy), time-domain methods (autoregressive modeling), frequency-domain approaches (PSD using the Welch method with a Hamming window (50% overlap), mu (8–12.5 Hz) and beta (13–30 Hz) powers, and their ratio (mu power/beta power)), time-frequency domain feature (discrete wavelet transform with Daubechies 4 wavelet function and the Fourier-based synchrosqueezing transform).	The classification methods investigated were the k-nearest neighbors, LDA, fine tree, Naive Bayes (Gaussian kernel) and SVM (Gaussian/quadratic kernel).<br><br>SPSS was used to analyze the data and the results statistically.	None	Not available
Ketola, E., Lloyd, C., Shuhart, D., Schmidt, J., Morenz, R., Khondker, A., & Imtiaz, M. (2022, January). Lessons Learned from the Initial Development of a Brain Controlled Assistive Device. In 2022 IEEE 12th Annual Computing and Communication Workshop and Conference (CCWC) (pp. 0580-0585). IEEE.	MI to control a mounted robotic arm with the aim of enable disabled people to perform basic lifting and moving tasks.	Emotiv EPOC X headset (reference electrodes on P3 and P4).	Six-axis DOFBOT robotic arm (commercially available for replicability), BLE 5.0 adapter, and a Raspberry Pi development board.<br>A modular design was preferred to allow further development.	In-built bandpass (0.2 - 45 Hz) and notch filtering.	Not specified	Not specified	Robotic arm movement.	Not available
Kevric, J., & Subasi, A. (2015). The impact of Mspca signal de-noising in real-time wireless brain computer interface system. Southeast Europe Journal of Soft Computing, 4(1).	Analyses with and without de-noising.	Mindwave Mobile Headset (one channel).	NA	Multiscale Principal Component Analysis (PCA) denoising.	EEG signal is decomposed into 16 sub-band signals using 4-level Wavelet Packet Decomposition (WPD) and six statistical features were extracted from each sub-band, generating 96 features in total.	Offline analysis with Matlab: SVM, Multi Layer Perceptron (MLP), decision tree, random forest, kNN, rotation forest.	None	Not available

Table S1. Detailed notes on the 84 reviewed papers. The notes are organized to provide a clear reference to the papers and to follow the core section of the review (Section 5), analyzing (i) field of applications, (ii) employed technologies, (iii) signal processing and analysis methodologies, (iv) BCI feedback, and (v) dataset information.

Reference	Application	Technologies (wireless, electrode type, storage, communication protocol)		Signal processing & analyses			Feedback	Dataset
		EEG related	other	pre-processing	feature engineering	classification and/or other analyses		
Khan, J., Bhatti, M. H., Khan, U. G., & Iqbal, R. (2019). Multiclass EEG motor-imagery classification with sub-band common spatial patterns. EURASIP Journal on Wireless Communications and Networking, 2019(1), 1-9.	Methodological for multi-class MI problem classification, i.e., MI task of neutral, left and right hand imagination of movement.	Emotiv EPOC+ for dry electrode technology testing: 10/20 international system, 14 electrodes (AF3, AF4, F7, F3, F4, F8, FC5, FC6, T7, T8, P7, P8, O1, O2) plus 2 reference electrodes.<br><br>Wet electrode device: non specified 8-channel (Fz, C3, Cz, C4, P7, P3, Pz, P8) EEG device.<br><br>Sampling rate Emotiv EPOC+: 128Hz.<br>Sampling rate wet device: 256Hz.	NA	Filter bank decomposition of EEG signal into sub-band.	Feature extraction through CSP and LDA, plus application of sequential backward floating selection (based on classifier best accuracy).	SVM, KNN, and Naïve Bayesian Parzen Window.	None	Not available
Khan, M. J., Hong, K. S., Naseer, N., & Bhutta, M. R. (2015, July). Motor imagery performance evaluation using hybrid EEG-NIRS for BCI. In 2015 54th Annual Conference of the Society of Instrument and Control Engineers of Japan (SICE) (pp. 1150-1155). IEEE.	Rehabilitation robot in an arm movement paradigm. Use combined EEG-NIRS for better signal classification.	g-MOBIlab + biosignal acquisition device. Six EEG electrodes (Ag/AgCl) were placed on the F3, F4, C3, C4, P3, and P4 brain location with reference to Cz.	Record simultaneously EEG and near-infrared spectroscopy data.	The alpha and beta bands were acquired by band-pass filtering the data between 8 - 12 Hz and 12 - 28 Hz.	PSD.	LDA classification.	Visual feedback.	Not available
Kim, H. H., & Jeong, J. (2019). Decoding electroencephalographic signals for direction in brain-computer interface using echo state network and Gaussian readouts. Computers in biology and medicine, 110, 254-264.	EEG decoder for user intention about 8 movement directions.	Emotiv Epoc + with 14 channels.	NA	ICA-based algorithm ADJUST to remove artifacts; band-pass (1 - 64 Hz) and (55 - 65 Hz) notch filter; autoregressive spectral analysis.	Not specified	Echo state network with genetic algorithm for parameter optimization and Gaussian readouts to create direction preferences.	None	Not available
Kong, W., Fu, S., Deng, B., Zeng, H., Zhang, J., & Guo, S. (2019). Embedded BCI Rehabilitation System for Stroke. Journal of Beijing Institute of Technology, 28(1), 35-41.	Stroke rehabilitation.	Emotiv.	EEG is transmitted to a Raspberry Pi processing unit through Bluetooth.	Not specified	FFT	Not specified	Visual feedback.	Not available
LaFleur, K., Cassady, K., Doud, A., Shades, K., Rogin, E., & He, B. (2013). Quadcopter control in three-dimensional space using a noninvasive motor imagery-based brain–computer interface. Journal of neural engineering, 10 (4), 046003.	Telepresence robotics: controling a flying robot in the three-dimensional physical space.	64-channel EEG Neuroscan Synamps 2.	EEG is sent regularly via wifi to the quadcopter robot to update its movement, while the quadcopter simultaneously acquires video and sends it back to the computer workstation.	EEG signals were sampled at 1000 Hz and filtered from DC 200 Hz before they were imported into BCI2000 with no spatial filtering.	Not specified	Electrode selection: C3 and C4 (BCI2000 offline analysis software). Quantify the performance of the system using a transfer rate metric for asynchronous real-world BCI.	Visual feedback was provided via a forward facing camera on the hull of the drone.	Not available
Li, Z., Yuan, Y., Luo, L., Su, W., Zhao, K., Xu, C., ... & Pi, M. (2019). Hybrid brain/muscle signals powered wearable walking exoskeleton enhancing motor ability in climbing stairs activity. IEEE Transactions on Medical Robotics and Bionics, 1 (4), 218-227.	Lower limb exoskeleton hybrid control achieved through an EEG and surface EMG (electromiography) based BCI to assist users in climbing stairs.	NeuroScan-NuAmps 40 channels digital EEG recording device, of which are used electrodes C3 and C4. Cz is chosen as the reference.<br><br>Sampling rate: 500Hz.	Delsys 2 channels EMG recording device, and a powered exoskeleton robot using Elmo driver.	Denoising: notch (50 Hz) and band-pass (5 - 30 Hz) filters.<br><br>Sliding window of 200 ms.	FFT to analyze time-frequency features.<br><br>CSP to extract EEG signal variance.	During the training phase, the EEG recognition accuracy is computed.<br><br>Back propagation NN (input layer, 2 hidden layers, and output layer - softmax) to recognize the imagery tasks. Mean square error is used to define the loss function.<br><br>NB: the EMG signals have been used only to adjust the exoskeleton gait.	Exoskeleton movement.	Not available

Table S1. Detailed notes on the 84 reviewed papers. The notes are organized to provide a clear reference to the papers and to follow the core section of the review (Section 5), analyzing (i) field of applications, (ii) employed technologies, (iii) signal processing and analysis methodologies, (iv) BCI feedback, and (v) dataset information.

**Reference**	**Application**	**Technologies (wireless, electrode type, storage, communication protocol)**		**Signal processing & analyses**			**Feedback**	**Dataset**
		**EEG related**	**other**	**pre-processing**	**feature engineering**	**classification and/or other analyses**		
Liao, L. D., Wu, S. L., Liou, C. H., Lu, S. W., Chen, S. A., Chen, S. F., ... & Lin, C. T. (2014). A novel 16-channel wireless system for electroencephalography measurements with dry spring-loaded sensors. IEEE Transactions on Instrumentation and Measurement, 63(6), 1545-1555.	Wireless and wearable multichannel EEG measurement system.	Custom EEG wearable device with adaptive cap based on the 10/20 international system.<br><br>The 16 electrodes (gold-coated) are composed by spring-loaded sensors with 8 probes that are able to provide a contact with the skin even having a great hair volume. A rubber pad is added for comfort.	Acquisition module: pre-amplifier unit, front-end ADC, microcontroller unit, wireless unit.<br><br>Adaptable cap with elastic material.	Not specified	Not specified	Verify signal quality: detect introduced distortion between wet and proposed dry technology.<br><br>A user weared at the same time wet and dry electrodes. The correlation between the sensors was accessed. A high correlation was detected between the wet and dry electrodes.<br><br>Positioning the electrodes in the sites Fpz, AFz, F8, F4, Fz, F3, F7, T7, T8, C4, Cz, C3, P4, Pz, P3, and Oz, a subject performed some measurements with eyes closed, eye blink, and teeth clenching.	None	Not available
Lin, B. S., Pan, J. S., Chu, T. Y., & Lin, B. S. (2016). Development of a wearable motor-imagery-based brain–computer interface. Journal of medical systems, 40 (3), 1-8.	Development of a wearable MI-based BCI with novel active comb-based electrodes.	4 active comb-shaped dry electrodes (C3, C4, Fz) located on a cap according to the 10/20 system.	NA	Common average reference to enhance the EEG signal.	Power difference in mu rhytm on C3 and C4. Mahalanobis distances between the left/right MI models derived from the EEG power difference.	Radial basis function neural network for event classification.	None	Not available
Lin, C. L., Chu, T. Y., Wu, P. J., Wang, C. A., & Lin, B. S. (2014, August). Design of wearable brain computer interface based on motor imagery. In 2014 Tenth International Conference on Intelligent Information Hiding and Multimedia Signal Processing (pp. 33-36). IEEE.	A wearable motor-imagery-based BCI system with the least number of EEG channels was developed. In this system, the wearable mechanical design and the wireless EEG acquisition module were designed for measuring real time EEG in daily life.	Custom wireless EEG acquisition module, and 3 novel dry EEG sensors (C3, C4, and Fz according to the 10/20 international system) to maintain good skin-electrode contact.	Bluetooth for communication.	Modified common average reference to enhance EEG components.	Normal EEG power in mu rhythm.	Multivariate distribution to discriminate motor imagery conditions (detect left hand MI).	None	Not available
Lisi, G., Hamaya, M., Noda, T., & Morimoto, J. (2016, May). Dry-wireless EEG and asynchronous adaptive feature extraction towards a plug-and-play co-adaptive brain robot interface. In 2016 IEEE International Conference on Robotics and Automation (ICRA) (pp. 959-966). IEEE.	Novel asynchronous adaptive BCI based on a dry-wireless headset, to trigger the movement of a lower limb exoskeleton robot by foot motor imagery.	Quick-20 (used F7, Fp1, Fp2, F8, F3, Fz, F4, C3, Cz, P8, P7, Pz, P4, T3, P3, O1, O2, C4, T4) dry-wireless headset (Cognionics, Inc) plus reference and ground electrodes.	Bluetooth for communication.<br><br>XoR lower limb exoskeleton robot.	Bandpass filtering (7 - 30 Hz).	CSP for feaure extraction plus feature adaptation and visualization.	Logistic regression classifier.	Exoskeleton control.	Not available
Lisi, G., Rivela, D., Takai, A., & Morimoto, J. (2018). Markov switching model for quick detection of event related desynchronization in EEG. Frontiers in neuroscience, 12, 24.	Propose a model for quick detection of ERD desynchronization in EEG. Possible applications in neurorehabilitation.	Quick-20 drywireless headset (Cognionics, Inc), 19 channels (F7, Fp1, Fp2, F8, F3, Fz, F4, C3, Cz, P8, P7, Pz, P4, T3, P3, O1, O2, C4, T4).<br><br>Sampling rate: 500 Hz.	NA	Filter (8 - 49 Hz), downsample at 100 Hz.	CSP.	Markov Switching Model to detect ERD elicited by MI.	None	Not available
Liu, Y., Habibnezhad, M., & Jebelli, H. (2021). Brain-computer interface for hands-free teleoperation of construction robots. Automation in Construction, 123, 103523.	Translate human brain signals into robotic commands in the context of construction sites.<br><br>Offline training and robotic manipulation (online) phases with conditions right, left and stop.	Wearable EEG system Emotiv Flex with 32 channels.<br><br>Sampling rate: 128 Hz.	Unmanned ground vehicle robot.<br><br>Design of a Robotic Operating System (ROS) environment.<br><br>Communication between devices through the TCP/IP communication protocol.	Offline training noise removal: bandpass filter (0.5 - 45 Hz) and discrete wavelet transform adaptive filter.<br><br>Online phase: bandpass filter (0.5 - 45 Hz).	Offline and online training: filter bank common spatial pattern to extract ERS and ERD spatial patterns.	Offline training: SVM as single predictive model. 108 SVM models were developed to classify the 108 training datasets. Afterwards, these models have been blended through an ensemble algorithm to provide a final classification.<br><br>Online phase: prediction through ensemble model.<br><br>They apply a kNN algorithm to counteract the possible loss of wirlessly transmitted data due to typical construction site interferences.<br><br>Develop a 2 command certainty evaluation algorithm to avoid errors (based on prior knowledge on the EEG input).	Robotic command execution.	Not available

Table S1. Detailed notes on the 84 reviewed papers. The notes are organized to provide a clear reference to the papers and to follow the core section of the review (Section 5), analyzing (i) field of applications, (ii) employed technologies, (iii) signal processing and analysis methodologies, (iv) BCI feedback, and (v) dataset information.

**Reference**	**Application**	**Technologies (wireless, electrode type, storage, communication protocol)**		**Signal processing & analyses**			**Feedback**	**Dataset**
		**EEG related**	**other**	**pre-processing**	**feature engineering**	**classification and/or other analyses**		
Lo, C. C., Chien, T. Y., Chen, Y. C., Tsai, S. H., Fang, W. C., & Lin, B. S. (2016). A wearable channel selection-based brain-computer interface for motor imagery detection. Sensors, 16 (2), 213.	Wearable channel selection-based (built in) BCI for MI detection.	Front-end EEG wearable device constituted by 8 retractable comb-shaped dry active electrodes to record the signal from hairy sites. The electrodes are F3, C3, P3, O1,<br>F4, C4, P4, and O2 (according to the 10/20 international system).<br><br>Wireless EEG acquisition module: analog multiplexer, analog CAR filter circuit, functional/reference switcher, EEG amplifier circuit, skin-electrode interface impedance test circuit, microprocessor, wireless transmission module.	Back-end host system (tablet provided with Windows 8 OS) communicating with the front-end through means of Bluetooth.<br><br>BCI monitoring program deployed with Microsoft C#.	Biquad tweaked Butterworth type filter to maintain the 8 - 13 Hz frequencies.	Average EEG power of the first 3 s computed for each training cycle (power baseline).<br><br>Selection of the channel providing the majority of information on ERD due to MI of left/right hand.	The ERD drives the MI detection.<br><br>EEG signal quality provided by the proposed electrodes are compared with standard wet ones: perform standard alpha rhythm experiment on channel Oz and verify that the performances of the 2 electrode types are similar; test on a SSVEP paradigm, which ensures that the peaks of flashing light is correctly captured; MI experiment with C3 (correlation between the electrode results).	None	Not available
Looned, R., Webb, J., Xiao, Z. G., & Menon, C. (2014). Assisting drinking with an affordable BCI-controlled wearable robot and electrical stimulation: a preliminary investigation. Journal of neuroengineering and rehabilitation, 11(1), 1-13.	Assess feasibility of assisting patiens affected by neurological disorders (upper extremities) through portable assistive technologies.<br><br>Provide means through which perform a real-life task (drinking water from glass).	Emotiv (128 Hz).<br><br>Open source software for EEG signal processing (BCI2000).	Custom robotic arm orthosis. EMPI300 functional electrical stimulator from DJO Global.<br><br>Bluetooth for communication.	Not specified	Not specified	Not specified	Cursor movement.<br><br>Robotic arm orthosis movement.	Not available
Mahmood, M., Kwon, S., Kim, H., Kim, Y. S., Siriaraya, P., Choi, J., ... & Yeo, W. H. (2021). Wireless Soft Scalp Electronics and Virtual Reality System for Motor Imagery‐Based Brain–Machine Interfaces. Advanced Science, 8(19), 2101129.	Wireless, real-time control of a virtual reality game.	Own made portable, wireless, soft scalp electronics with 6 EEG channels: Fz, C5, C3, C4, C6, and POz.	Composed of: 1) multiple flexible microneedle electrodes for mounting on the hairy scalp, 2) laser-machined stretchable and flexible interconnects, 3) a low-profile, flexible circuit. Inclusion of a virtual reality (VR) component. EEG data were recorded using a custom application running on an Android Tablet (Samsung Galaxy Tab S4), using Bluetooth Low Energy wireless communication.	3rd-order Butterworth bandpass filter (4 - 33 Hz) and segmented into 4 s windows with a 50% overlap between consecutive windows.	PSD estimation using the Welch method.	SVM and CNN classifiers (4 class).	Visual feedback.	Available upon request
Mattia, D., Pichiorri, F., Colamarino, E., Masciullo, M., Morone, G., Toppi, J., ... & Molinari, M. (2020). The Promotoer, a brain-computer interface-assisted intervention to promote upper limb functional motor recovery after stroke: a study protocol for a randomized controlled trial to test early and long-term efficacy and to identify determinants of response. BMC neurology, 20(1), 1-13.	Study protocol. Propose a Promotoer system: EEG-based BCI for post stroke rehabilitation.	g.MOBIlab (g.tec medical engineering GmbH Austria).	The Promoter is also equipped with a computer, a screen for the therapist feedback (for the EEG activity and EMG activity monitoring) and screen for the ecological feedback to the participant.	Not specified	Not specified	Not specified	Ecological feedback.	Not available
Mitocaru, A., Poboroniuc, M. S., Irimia, D., & Baciu, A. (2021, October). Comparison Between Two Brain Computer Interface Systems Aiming to Control a Mobile Robot. In 2021 International Conference on Electromechanical and Energy Systems (SIELMEN) (pp. 1-5). IEEE.	Control of a mobile robotic platform using the Steady State Visually Evoked Potential and the Motor Imagery methods.	They used both wired (g.USBamp and 8 g.SAHARA dry active electrodes) and wireless EEG. The wireless device is a 32-channel g.Nautilus.	Lynxmotion A4wd1 mobile robotic platform.	Not specified	Not specified	Not specified	Control of a mobile robot.	Not available
Mladenov, T., Kim, K., & Nooshabadi, S. (2012, June). Accurate motor imagery based dry electrode brain-computer interface system for consumer applications. In 2012 IEEE 16th International Symposium on Consumer Electronics (pp. 1-4). IEEE.	BCI consumer grade application based on 2 dry active electrodes.	Emotiv EPOC chosing only C3 and C4 electrodes to conduct a MI experiment.<br><br>Sampling rate: 128 Hz.	Bluetooth for communication.	Components above 40 Hz filtered out.<br><br>CAR plus baseline, zero-mean, and unit variance normalization.	FFT and (Infinite Impulse Response) IIR tuned to specific rhytms to extract ERD and ERS information.	Not specified	Object move across the screen depending on the provided command.	Not available

Table S1. Detailed notes on the 84 reviewed papers. The notes are organized to provide a clear reference to the papers and to follow the core section of the review (Section 5), analyzing (i) field of applications, (ii) employed technologies, (iii) signal processing and analysis methodologies, (iv) BCI feedback, and (v) dataset information.

Reference	Application	Technologies (wireless, electrode type, storage, communication protocol)		Signal processing & analyses			Feedback	Dataset
		EEG related	other	pre-processing	feature engineering	classification and/or other analyses		
Mwata-Velu, T. Y., Ruiz-Pinales, J., Rostro-Gonzalez, H., Ibarra-Manzano, M. A., Cruz-Duarte, J. M., & Avina-Cervantes, J. G. (2021). Motor imagery classification based on a recurrent-convolutional architecture to control a hexapod robot. Mathematics, 9(6), 606.	Real-time embedded MI-based BCI system applied to hexapod robot for testing locomotion imagery commands.	Emotiv EPOC+ (using F3, F4, FC5, and FC6).	Altera SoCKit development board and a hexapod robot.	Low-pass filter (85 Hz cutoff), bandpass filter (0.16 - 43 Hz), band-stop filter (50 - 60 Hz). Mu rhythm considered for further analysis.	CNN-LSTM architecture to conduct both feature extraction and data classification.	CNN-LSTM architecture to conduct both feature extraction and data classification.<br><br>Qualitative and quantitative analysis.	Movement of a hexapod robot.	Custom dataset and public dataset https://www.nature.com/articles/sdata2018211 for comparison.
Parikh, D., & George, K. (2020, November). Quadcopter Control in Three-Dimensional Space Using SSVEP and Motor Imagery-Based Brain-Computer Interface. In 2020 11th IEEE Annual Information Technology, Electronics and Mobile Communication Conference (IEMCON) (pp. 0782-0785). IEEE.	Mathematical modeling and numerical simulation of a quadcopter and using MI and SSVEP based BCI.	Emotiv EPOC+ (and EmotivPro for signal processing).<br>Employed electrodes: C1, Cz, C2, O1, Oz, O2 with Fz as reference and ground at AFz.	DJI Flight Simulator.	Low-pass filter (40 Hz cutoff).	Not specified	Not specified	Virtual quadcopter controlling.	Not available
Paszkiel, S., & Dobrakowski, P. (2020). Brain–computer technology-based training system in the field of motor imagery. IET Science, Measurement & Technology, 14 (10), 1014-1018.	Training system for MI upper limb generation.	Emotiv Epoc Flex using electrodes C3, FC5, C4, FC8, T7, P3, Oz, Cpz, P4, T8, FCz, F4, Cz for the training session and electrodes C3, C4, Cz, CP{1,2,5,6}, FC {1,2,5,6,z} for the monitoring session.	MyoWare muscle sensor device.	Common average reference and Laplacian filtering.	FFT.	Not specified	None	Not available
Permana, K., Wijaya, S. K., & Prajitno, P. (2019, November). Controlled wheelchair based on brain computer interface using Neurosky Mindwave Mobile 2. In AIP Conference Proceedings (Vol. 2168, No. 1, p. 020022). AIP Publishing LLC.	Wheelchair control through MI-based BCI.	NeuroSky Mindwave Mobile 2 headset using FP1 electrode only.	Bluetooth transmission to wheelchair micro-controller.	Not specified	Not specified	Not specified	Wheelchair control.	Not available
Peterson, V., Galván, C., Hernández, H., & Spies, R. (2020). A feasibility study of a complete low-cost consumer-grade brain-computer interface system. Heliyon, 6(3), e03425.	Applicability and feasibility study on OpenBCI devices in real-life out-of-laboratory environments.<br><br>Encourage motor function (affordable BCI) rehabilitation at home.	EEG signal recording through OpenBCI Cyton + Daisy Module and Electrocap System II (Fz, F3, F4, F7, F8, Cz, C3, C4, T3, T4, Pz, P3, P4, T5, T6 wet electrodes, reference and ground on earlobes, 125 Hz sampling rate).<br><br>Recording through OpenViBE free software (running on Linux OS).	EMG monitoring through OpenBCI Ganglion (4 channels, 200 Hz sampling rate) board and Myoware sensors.<br><br>Recording through OpenBCI GUI free software (running on Windows OS).<br><br>USB Dongle for wirless transmission.	During EEG signal acquisition: 3rd order Butterworth filter (0.5 - 45 Hz).<br>Post processing: backward-forward bandpass 5th order Butterworth filter (1 - 40 Hz).<br><br>IIR notch filter (50 Hz) and 5th order Butterworth high-pass filter (10 Hz) to remove noise from EMG signal.	Penalized time-frequency band common spatial pattern (PTFBCSP) for MI detection.<br><br>Generalized sparse discriminant analysis is used for both feature selection and classification.	Generalized sparse discriminant analysis is used for both feature selection and classification.<br><br>The analysis was conducted both online and offline to evaluate the performance of the system.<br><br>The motor imagery ability of a single subject has been accessed through the KVIQ-10 questionnaire.	None	See: https://github.com/vpeterson/MI-OpenBCI
Peterson, V., Wyser, D., Lambercy, O., Spies, R., & Gassert, R. (2019). A penalized time-frequency band feature selection and classification procedure for improved motor intention decoding in multichannel EEG. Journal of neural engineering, 16(1), 016019.	Novel approach for motor intention detection enhancement by automatic selection of subject-specific spatio-temporal-spectral features.<br><br>Scenario: BCI-based functional hand motor rehabilitation.	eego(TM) rt Ant Neuro.	NA	Penalized time-frequency band CSP: raw signal decomposed in T temporal windows, which are split in F frequency bands, spatial features extracted through CSP.<br><br>Optimal feature selection and classification contemporaneally performed through generalized sparse LDA.			None	Own dataset not available + BCI competition III dataset IVa and BCI competition IV dataset IIb.

Table S1. Detailed notes on the 84 reviewed papers. The notes are organized to provide a clear reference to the papers and to follow the core section of the review (Section 5), analyzing (i) field of applications, (ii) employed technologies, (iii) signal processing and analysis methodologies, (iv) BCI feedback, and (v) dataset information.

Reference	Application	Technologies (wireless, electrode type, storage, communication protocol)		Signal processing & analyses			Feedback	Dataset
		EEG related	other	pre-processing	feature engineering	classification and/or other analyses		
Priyatno, S. B., Prakoso, T., & Riyadi, M. A. (2022). Classification of motor imagery brain wave for bionic hand movement using multilayer perceptron. Sinergi, 26(1), 57-64.	Bionic hand control application.	Muse headband 2: 10/20 international system, 4 goldplated cup bipolar electrodes (TP9, AF7, AF8, TP10). The Fpz electrode is used only as a reference.	Bluetooth connection with a Raspberry Pi.	Not specified	Mean absolute power extracted through FFT application.	Multilayer perceptron (ReLU activation function, Adam optimizer) to classify 5 conditions: release, key grip, finger point, open precision, spoon hold.	Bionic hand movement.	Not available
Quiles, E., Suay, F., Candela, G., Chio, N., Jiménez, M., & Álvarez-Kurogi, L. (2020). Low-cost robotic guide based on a motor imagery brain–computer interface for arm assisted rehabilitation. International journal of environmental research and public health, 17 (3), 699.	MI-BCI controlled low-cost robotic guide for assisted rehabilitation of arm movements.	Enobio 8: medically certified 8 dry/wet electrodes wearable device with flexible positioning of the electrodes. The authors used the F3, F4, C3, Cz, C4, T7, T8, and Pz according to the 10/20 international standard. Moreover, reference and ground were placed on the earlobe.<br><br>Sampling rate: 500Hz.<br><br>BCI2000 for rehabilitation guide control.	Metallic aluminum and 3D printed rehabilitation guide allowing rehabilitaiton of different arm movements. Gears are present to allow movements and rotations plus a motor controlled through a driver or H-bridge (BD6231F-E2). Through a NI-6210 acquisition card, LabVIEW controls the device movent. Battery alimented.<br><br>Wristbands adapted to the single subject.<br><br>Bluetooth for communication.	Bandpass filtering (2 - 100Hz) and notch filtering (50 Hz).<br><br>SNR improvement through Laplacian filtering on C3, Cz, and C4.	Estimation of sensorimotor rhythms and power spectrum to finally obtain the maximum entropy on C3, Cz, C4.	t-test comparison of experiment 1 and 2 results: tha action-action strategy of experiment 2 seems to provide better performances in terms of BCI control.<br><br>95% of the subjects seem to have had a good experience according to a questionnaire, especially the wearable EEG device did not provide discomfort.<br><br>Limitation: the participation of the experimentation was mandatory for one of the authors' course completion.	Rehabilitation guide movement.	Not available
Riyadi, M. A., Setiawan, I., & Amir, A. (2021, September). EEG Multiclass Signal Classification Based on Subtractive Clustering-ANFIS and Wavelet Packet Decomposition. In 2021 International Conference on Electrical and Information Technology (IEIT) (pp. 81-86). IEEE.	Left/right hand MI with EEG headband to promote BCI for disabled people.	Muse headband 2: 10/20 international system, 4 goldplated cup bipolar electrodes (TP9, AF7, AF8, TP10).	NA	ICA for noise removal and Z-score normalization.	Wavelet Packet Decomposition (db4), using signals at alpha and beta rhythms on channels AF7 and AF8. The authors consider statistical features (e.g., range and standard deviation of signal amplitude, signal spectrum strength values).	ANFIS method and the Subtractive Clustering.	None	Not available
Rodriguez-Ugarte, M. D. L. S., Iáñez, E., Ortiz-Garcia, M., & Azorín, J. M. (2018). Effects of tDCS on real-time BCI detection of pedaling motor imagery. Sensors, 18(4), 1136.	Online BCI that distinguishes between two different cognitive neural states (relax and pedaling MI) to strengthen the cortical excitability over M1 and the cerebro-cerebellar pathway by means of transcranial direct current stimulation (tDCS) configuration and thus better detect lower limb motor imagery in real time.	StarStim R32 (Neuroelectrics). Device connected through a USB isolator to the computer. Based on the 10/10 system, the EEG signals were acquired from 30 channels (P7, P4, CZ, PZ, P3, P8, O1, O2, C2, C4, F4, FP2, FZ, C3, F3, FP1, C1, OZ, PO4, FC6, FC2, AF4, CP6, CP2, CP1, CP5, FC1, FC5, AF3, and PO3) with two reference electrodes (CMS and DRL) at a frequency rate of 500 Hz.	NA	4th-order Butterworth high-pass filter with cut-off frequency 0.05 Hz. Notch filter(50 Hz). 4th-order Butterworth low-pass used to eliminate the power line interference at 50 Hz. 4th order Butterworth low-pass filter with cut-off frequency of 45 Hz. Laplacian spacial filter.<br><br>Only Cz, CP1, CP2, C1, C2, C3, C4, FC1 and FC2 were considered.	Nine features (the powers at each electrode optimal frequency).	SVM classifier.	Real-time positive feedback about performance using the output from the BCI.	Not available
Rodríguez-Ugarte, M., Angulo-Sherman, I. N., Iáñez, E., Ortiz, M., & Azorín, J. M. (2017, November). Preliminary study of pedaling motor imagery classification based on EEG signals. In 2017 International Symposium on Wearable Robotics and Rehabilitation (WeRob) (pp. 1-2). IEEE.	Preliminary study: comparison of SVM and LDA classifiers on delta, mu and beta frequency bands.	StarStimR32.	NA	Bandpass (0.05 - 30 Hz) filter.<br><br>ICA to remove artifacts (e.g. blinking).	Not specified	SVM and LDA to detect pedaling vs rest.	None	Not available
Shajil, N., Mohan, S., Srinivasan, P., Arivudaiyanambi, J., & Arasappan Murrugesan, A. (2020). Multiclass classification of spatially filtered motor imagery EEG signals using convolutional neural network for BCI based applications. Journal of Medical and Biological Engineering, 40 (5), 663-672.	Multiclass CNN classification for spatial filtered EEG-based MI-BCIs.	Enobio 20 wireless system, dry unipolar electrodes. The authors used the F3, Fz, F4, FC5, FC1, FC2, FC6, C3, C1, CZ, C2, C4, CP5, CP1, CP2, and CP6 electrodes. A reference electrode was placed on the right ear lobe.<br><br>Sampling rate: 500Hz.	Acquisition through OpenViBE.	Bandpass filtering (0 - 125 Hz) and notch filtering (50 Hz) during recording.<br><br>Channel selection: C3, C1, Cz, C2, C4.<br><br>Bandpass filtering through Butterworth 5th-order filtering: (i) considering 1 - 100 Hz, (ii) considering 8 - 30 Hz.<br>The spatial resolution was increased through CSP.	Not specified	Data representation in a 2D image considering the spectrogram of the filtered signals.<br><br>Data augmentation through image flipping.<br><br>A CNN was used for classification of binary conditions (left/right hand fisting MI) and of multi-class problem (left/right hand fisting,both hands fisting or both foot tapping MI).	None	Not available

Table S1. Detailed notes on the 84 reviewed papers. The notes are organized to provide a clear reference to the papers and to follow the core section of the review (Section 5), analyzing (i) field of applications, (ii) employed technologies, (iii) signal processing and analysis methodologies, (iv) BCI feedback, and (v) dataset information.

**Reference**	**Application**	**Technologies (wireless, electrode type, storage, communication protocol)**		**Signal processing & analyses**			**Feedback**	**Dataset**
		**EEG related**	**other**	**pre-processing**	**feature engineering**	**classification and/or other analyses**		
Shajil, N., Sasikala, M., & Arunnagiri, A. M. (2020, October). Deep learning classification of two-class motor imagery EEG signals using transfer learning. In 2020 International Conference on e-Health and Bioengineering (EHB) (pp. 1-4). IEEE.	Exploit transfer learning for MI based BCI considering 3 pre-trained CNN models.<br><br>The analysis uses a custom dataset and the BCI competition IV dataset 2a.	Wireless EEG system provided with dry electrodes (C3, C4, Cz) plus a reference electrode placed on the right earlobe.	NA	Signal sampling to 500 Hz and notch filtered (50 Hz).<br>5th order IIR Butterworth filter (8 - 30 Hz) application.<br><br>Short time Fourier Transform is applied to obtain 2D images representing the time and frequency components of the signals. The channel images are stacked togheter and then passed as inputs to the DL models.	AlexNet, ResNet50, and InceptionV3 are used as pre-trained CNN models.	The same (feature engineering) architectures are used to classify the left/right hand MI conditions, considering a transfer learning strategy.	None	Own dataset not available and BCI competition IV dataset 2a.
Simon, C., & Ruddy, K. L. (2022, February). A wireless, wearable Brain-Computer Interface for neurorehabilitation at home; A feasibility study. In 2022 10th International Winter Conference on Brain-Computer Interface (BCI) (pp. 1-6). IEEE.	Study the feasibility of employing EEG wearable technologies in home environments.	16 channel EEG cap with dry contact electrodes (Cumulus, Belfast, UK). Reference (disposable) electrodes were placed on the mastoids.	Bluetooth for communication.<br><br>OpenViBE for Graz-BCI like protocol execution.	Butterworth (8 - 30 Hz), 5th order bandpass ripple of 1Hz.	Not specified	CSP and LDA for binary condition classification.	Bar going up or down depending by the given command.	Not available
Tang, X., Li, W., Li, X., Ma, W., & Dang, X. (2020). Motor imagery EEG recognition based on conditional optimization empirical mode decomposition and multi-scale convolutional neural network. Expert Systems with Applications, 149, 113285.	EEG-MI for intelligent wheelchair BCI system: decode left and right hand motor imagination to provide a left or right wheelchair movement.	Emotiv EPOC+.<br>Employed electrodes: F3, F4, FC5, FC6, T7, T8 plus 2 reference electrodes.<br><br>Sampling rate: 128Hz.	Intelligent wheelchair.	Denoising: abnormal sample removal, baseline correction, bandpass filtering (8 - 30 Hz).<br><br>Conditional empirical mode decomposition for single electrode signal denoising, based on the correlation between the original signal and the obtained intrinsic mode functions.	Time-domain feature extraction through 1D convolution plus between channel information to capture ERD/ERS.	Own dataset: each of the 5 subject data divided in training and test sets according to the 4:1 ratio.<br><br>BCI Competition IV dataset 2b for performance evaluation: the proposed method has better results in respect to benchmark algorithms.<br><br>1DCNN, 2DCNN and !D multi scale CNN (1DMSCNN) for EEG signal classification. The 1DMSCNN achieves the best performances.<br><br>Average signal accuracy and standard error computation plus ROC and AUC.	On final online experiment (after own dataset and BCI competition IV testing): wheelchair straight/left/right movement.	Only the BCI Competition IV dataset 2b is available online.
Tariq, M., Trivailo, P. M., & Simic, M. (2018). Motor imagery based EEG features visualization for BCI applications. Procedia computer science, 126, 1936-1944.	Application to BCI robotic foot movement considering the use of OpenViBE.	EEG neurofeedback (24-channel) BrainMaster Discovery 24E.	Robotic foot.<br><br>OpenViBE for visualization of data in offline mode.	Bandpass filtered using 5th-order Butterworth filter (8 - 11 Hz) for mu and (12 - 30 Hz) for beta rhythms.	Band power features. ERD and ERS patterns and spectral analysis based on FFT.	No classification: evaluation of OpenVibe for offline/online analysis.	None	Not available
Tiwari, S., Goel, S., & Bhardwaj, A. (2020, July). Machine learning approach for the classification of EEG signals of multiple imagery tasks. In 2020 11th International Conference on Computing, Communication and Networking Technologies (ICCCNT) (pp. 1-7). IEEE.	Classification of multiple imagery tasks.	Muse headband 2: 10/20 international system, 4 goldplated cup bipolar electrodes (TP9, AF7, AF8, TP10). The Fpz electrode is used only as a reference.	NA	In-built notch filtering.	FFT and DWT to extract PSD of delta, theta, alpha and beta rhythms.	Logistic Regression and Quadratic Discriminant Analysis.	None	Not available
Triana Guzmán, N., Orjuela-Cañón, Á. D., & Jutinico Alarcon, A. L. (2019, October). Incremental training of neural network for motor tasks recognition based on brain-computer interface. In Iberoamerican Congress on Pattern Recognition (pp. 610-619). Springer, Cham.	Incremental training proposal for BCI systems. Comparison with traditional cross-validation techniques.	Nautilus system (g.Tec medical engineering GmbH, Schiedlberg, Austria) at 250 Hz.	NA	C3, Cz, and C4 selected.<br><br>Butterworth bandpass filter (0.5 - 60 Hz).	Spectral power.	MLP classification of left/right hand and resting state.	None	Not available

Table S1. Detailed notes on the 84 reviewed papers. The notes are organized to provide a clear reference to the papers and to follow the core section of the review (Section 5), analyzing (i) field of applications, (ii) employed technologies, (iii) signal processing and analysis methodologies, (iv) BCI feedback, and (v) dataset information.

Reference	Application	Technologies (wireless, electrode type, storage, communication protocol)		Signal processing & analyses			Feedback	Dataset
		EEG related	other	pre-processing	feature engineering	classification and/or other analyses		
Venot, T., Corsi, M. C., Saint-Bauzel, L., & de Vico Fallani, F. (2021, November). Towards multimodal BCIs: the impact of peripheral control on motor cortex activity and sense of agency. In 2021 43rd Annual International Conference of the IEEE Engineering in Medicine & Biology Society (EMBC) (pp. 5876-5879). IEEE.	Analyses of the link between sense of agency and motor imagination.	Enobio 8: medically certified 8 dry/wet electrodes wearable device with flexible positioning of the electrodes. The authors used the Cz, FC1, C1, CP1, P3, CP5, C3, and FC5 electrodes.<br><br>Sampling rate: 500Hz. Considering a bandwidth of 0-60Hz.	Tobii Pro X3 eye tracking device plus Kinect v1.<br><br>Projector for virtual robot visualization.	ICA through EEGLAB toolbox for MATLAB.	PSD estimation through Welch's method, considering the alpha and beta rhythms.	Permutation student t-test statistical analyses through Brainstorm on the different conditions of motor activity, eye gaze and no activity.<br><br>The active conditions revealed a higher sense of agency, but there were no significant differences between these two conditions. The authors observed that gaze control affect the motor cortex activity. Similar areas involved with motor imagery seemed to be solicited.<br><br>The authors set the experiment considering only a right-handed configuration and suggest it as a limitation of their work.	Virtual robotic arm movement on screen.	Not available
Verma, P., Heilinger, A., Reitner, P., Grünwald, J., Guger, C., & Franklin, D. (2019, October). Performance investigation of brain-computer interfaces that combine EEG and fNIRS for motor imagery tasks. In 2019 IEEE International Conference on Systems, Man and Cybernetics (SMC) (pp. 259-263). IEEE.	Investigate the performance of BCIs by combining EEG and fNIRS modalities.	Hybrid EEG-fNIRS device: g. Nautilus fNIRS (g.tec) with 15 EEG gel electrodes.	NA	Bandpass filter (0.5 - 30 Hz) and notch filter (48 - 52 Hz).	EEG and fNIRS processing offline with Matlab and g.BSanalyze (g. tec). EEG signals filtered in the mu and beta band using a 4th order recursive Butterworth bandpass filter from 8 - 25 Hz.	CSP and PCA for dimensionality reduction.<br><br>LDA as meta classifier (different combinations of EEG and fNIRS modalities).	None	Not available
Vourvopoulos, A., Jorge, C., Abreu, R., Figueiredo, P., Fernandes, J. C., & Bermudez i Badia, S. (2019). Efficacy and brain imaging correlates of an immersive motor imagery BCI-driven VR system for upper limb motor rehabilitation: A clinical case report. Frontiers in human neuroscience, 13, 244.	Case study for the assessment of the efficacy of EEG-based MI-BCI-VR system for the rehabilitation of upper limbs after stroke.	NeuRow BCI-VR system EEG data acquisition through Enobio 8, medically certified 8 dry/wet electrodes wearable device with flexible positioning of the electrodes. The authors used the FC5, FC6, C1, C2, C3, C4, CP5, CP6 electrodes.<br><br>Sampling rate: 500Hz.	Bluetooth for communication. Data streaming through Reh@Panel for virtual environment control.<br><br>Visual feedback through Oculus Rift DK1 HMD.<br><br>Vibrotactile feedback through a custom module made of a pair of tubes that the participant had to take into his hands, vibrating through the use of an Arduino Mega 2560 board and vibrating motors.<br><br>NeuRow: first-person self-paced (no cuing) BCI game to increase ecological validity. Based on Unity.	Online analysis: bandpass filtering (8 - 30 Hz), maximization of inter-signal differences through CSP.<br><br>Offline analysis: EEGLAB toolbox usage for high-pass filtering at 1 Hz, line noise and harmonics removal (50 Hz). Bad channel rejection, data re-referenced to the average. Eye blinking and motion artifacts removal through ICA (ICLabel plug-in).	Offline analysis: PSD estimation through Welch's method for alpha, beta, theta, and gamma rhythms. The alpha PSD was extracted from an initial resting-state phase. ERD/ERS were computed across the mu and beta rhythms on the C3 and C4 electrodes.<br>The lateralization index was accessed.	LDA classifier for the left/right hand conditions discrimination.<br>Its output was used as input to an adaptive performance engine.<br><br>Notice that the patient was also submitted to fMRI controls.<br><br>Results show improvements and recovery of motor functions.<br><br>Limitation: a single participant and the absence of a control group. There is also a great variability in the band power, which may be not completely representative of the final results.	Visual feedback through VR, Auditory feedback in game, Vibrotactile feedback for hand stimulation.	Available upon request
Vourvopoulos, A., Niforatos, E., & Giannakos, M. (2019, September). EEGlass: An EEG-eyeware prototype for ubiquitous brain-computer interaction. In Adjunct proceedings of the 2019 ACM international joint conference on pervasive and ubiquitous computing and proceedings of the 2019 ACM international symposium on wearable computers (pp. 647-652).	Preliminary study on an innovative EEG device integrated to eyeglasses for ubiquitous Human Machine Interaction (HMI), tested on a single subject.	Custom EEG-eyeware (EEGlass): plastic frame adaptable to Google Glass HMD, embedded EEG system Cyton Biosensing Board (OpenBCI). The electrodes were placed on TP9 and TP10 according to the 10/10 international system. Another electrode was placed on the glabella and 2 electrodes were used as reference and ground touching the opposite sides of the nose.<br><br>Enobio 8: medically certified 8 dry/wet electrodes wearable device with flexible positioning of the electrodes. The authors used Fpz, C3, C4, and Pz. Moreover, reference and ground were placed on the right earlobe.	Bluetooth for communication.<br><br>OpenVibe for simultaneous signal acquisition from the standard and custom devices.	High pass filtering at 1 Hz (baseline drift removal), line noise and harmonic removal (50 Hz).	PSD estimation through Welch's method on delta, theta, alpha, and beta rhythms.<br>ERD/ERS extraction on the C3 and C4 electrodes for the standard device, and on the TP9 and TP10 electrodes for the custom one.	Verify if the results obtained through a "standard" device can be approximated by the proposed solution.<br><br>t-test comparison of average ERD taken from lateral electrodes of the 2 devices (TP9 vs C3, and TP10 vs C4) verifies that there is a detection of motor movement in both devices with different amplitudes and spatial resolution.	None	Not available (preliminary study on a single subject)

Table S1. Detailed notes on the 84 reviewed papers. The notes are organized to provide a clear reference to the papers and to follow the core section of the review (Section 5), analyzing (i) field of applications, (ii) employed technologies, (iii) signal processing and analysis methodologies, (iv) BCI feedback, and (v) dataset information.

**Reference**	**Application**	**Technologies (wireless, electrode type, storage, communication protocol)**		**Signal processing & analyses**			**Feedback**	**Dataset**
		**EEG related**	**other**	**pre-processing**	**feature engineering**	**classification and/or other analyses**		
Wang, H., & Bezerianos, A. (2017). Brain-controlled wheelchair controlled by sustained and brief motor imagery BCIs. Electronics Letters, 53(17), 1178-1180.	Wheelchair precise control through MI based BCI.	3D printed wireless 24 dry sensors EEG headset acquisition system (Cognionics, Inc., USA). Reference channels placed on the mastoids.<br><br>Sampling rate: 250 Hz.	Robotic wheelchair.	Bandpass filtering (0.5 - 30 Hz) directly from Cognionics acquisition SW.<br><br>Spatial filtering through Common Average Reference (CAR) and band pass filtering (8 - 12 Hz) to extract the mu rhythm.	CSP for future extraction.	SVM to classify left/right hand and both hands imagination.	Wheelchair movement.	Not available
Wu, S. L., Liu, Y. T., Chou, K. P., Lin, Y. Y., Lu, J., Zhang, G., ... & Lin, C. T. (2016, July). A motor imagery based brain-computer interface system via swarm-optimized fuzzy integral and its application. In 2016 IEEE International Conference on Fuzzy Systems (FUZZ-IEEE) (pp. 2495-2500). IEEE.	Enahnce BCI performance, usability and safety through a novel classification technique.	Custom wireless device with dry electrodes (C3, Cz, C4). The reference electrodes were placed on the earlobes.	Bluetooth for communication.	Filter bank (1 - 30 Hz) to extract the delta, theta, alpha and beta rhythms.	Employ sub-band CSP to extract features from each of the rhythms.	Classification (left/right hand MI) is performed on each of the obtained feature sets through multiple LDA. Afterwards, the classifiers are combined through fuzzy integration (Sugeno and Chouquet integrals). PSO is employed to assign confidence levels to the classifiers (ROC curve optimization).	Accuracy reported on screen as a bar.<br><br>Robotic arm grasps a glass on the left or right depending on a free given command.	Not available
Xu, B., Li, W., He, X., Wei, Z., Zhang, D., Wu, C., & Song, A. (2020). Motor imagery based continuous teleoperation robot control with tactile feedback. Electronics, 9(1), 174.	BCI-based continuous teleoperation robot control system with tactile feedback.	g.MOBIlab (g.tec portable acquisition system API for g. USBamp): C3, FC3, CP3, C5, C4, FC4, CP4, and C6.<br><br>Sampling rate: 256 Hz.	Wireless portable acquisition. EEG signals acquired from the amplifier using the BCI2000 software by Bluetooth.<br><br>Robotic arm moved through the control signal in real time.	CAR filter.	Not specified	Autoregressive (AR) model.	Visual and vibrotactile feedback.	Not available
Yang, B., Tang, J., Guan, C., & Li, B. (2018, July). Motor imagery EEG recognition based on FBCSP and PCA. In International Conference on Brain Inspired Cognitive Systems (pp. 195-205). Springer, Cham.	Propose a Filter Bank CSP algorithm, avoiding the problem of manually adjusting the frequency range selected caused by individual differences when using traditional CSP algorithm.	g.Nautilus-8 wireless (dry electrodes).	NA	Band-pass filtering (0.05 - 200 Hz).	CSP for feature extraction and ICA for feature vector dimensionality reduction.	SVM and KNN. Classification on own dataset and on BCI competition iV dataset 1, subject c,d,e,g.<br>Tasks considered: left and right hand MI.	None	Not available
Yang, D., Nguyen, T. H., & Chung, W. Y. (2020). A Synchronized Hybrid Brain-Computer Interface System for Simultaneous Detection and Classification of Fusion EEG Signals. Complexity, 2020.	Synchronized hybrid BCI system for simultaneous detection and classification of fusion EEG signals.	High-density wireless EEG headset (Cognionics Inc.). Employed electrodes: C3 and C4.<br><br>Sampling rate: 500Hz.	NA	Bandpass filtering (1 - 50 Hz) on the signal plus to extract mu rhythm (8 - 13 Hz) and SSVEP signals (6.6 - 9.6 Hz).<br><br>FastICA to extract the signals.	Features in the time and frequency domain with the wavelet transform.	CNN to detect multiple mental tasks.	None	Available upon request
Yusoff, M. Z., Mahmoud, D., Malik, A. S., & Bahloul, M. R. (2018). Discrimination of four class simple limb motor imagery movements for brain–computer interface. Biomedical Signal Processing and Control, 44, 181-190.	Simple limb MI for the development of a MI-based BCI.	Enobio 8: medically certified 8 dry/wet electrodes wearable device with flexible positioning of the electrodes, according to the 10/20 international system. Employed electrodes: C3, Cz, and C4.<br><br>Sampling rate: 500Hz.	NA	Reduce SNR: bandpass filtering (0.5 - 30 Hz) using a 3rd-order Butterworth filter, and CAR filter.<br><br>Reduce data dimensionality: selection of C3, Cz, and C4 channels plus data segmentation.	Compare energy, entropy, and absolute values of alpha and beta rhythms derived from (i) discrete wavelet transform application (Symlets 5) or (ii) Empirical Mode Decomposition (EMD) computation.	Use Feed Forward Back Propagation Neural Network to predict 4 motor imagery tasks (left/right hand/foot MI). The NN had 1 input, 1 hidden and 1 output layer. The authors tested different neuron sizes and activation functions.<br><br>The EMD extracted features allowed better performances of the BCI.<br>The discrimination of left/right foot MI was particularly difficult.<br>Inter-subject variability was observed.	None	Not available
Zhang, S., Yuan, S., Huang, L., Zheng, X., Wu, Z., Xu, K., & Pan, G. (2019). Human mind control of rat cyborg's continuous locomotion with wireless brain-to-brain interface. Scientific reports, 9(1), 1-12.	Brain machine interface from the human brain to a rat implanted with microelectrodes, which integrated EEG-based MI and brain stimulation to realize human mind control of the rat continuous locomotion.	Emotiv Epoc.	Recorded wirelessly transmitted to a host computer through Bluetooth and further processed with Emotiv SDK.	Not specified	Power spectrum and CSP.	Not specified	Human mind control of the rat's continuous locomotion.	Available upon request
Zhang, X., Yao, L., Sheng, Q. Z., Kanhere, S. S., Gu, T., & Zhang, D. (2018, March). Converting your thoughts to texts: Enabling brain typing via deep feature learning of eeg signals. In 2018 IEEE international conference on pervasive computing and communications (PerCom) (pp. 1-10). IEEE.	Motor imagery BCI system for typing. The testing considers both a public available from literature and custom dataset.	Wired EEG public dataset and wireless EEG.<br>The wireless EEG is an Emotiv Epoc + with 14 channels.	TCP for signal transmission.	Not specified	A novel hybrid deep neural network that combines the benefits of both Convolutional Neural Networks (CNNs) and Recurrent Neural Networks (RNNs).	Quantify intent in EEG signals through Pearson Correlation Coefficient.<br><br>Extreme Gradient Boosting classifier for intent recognition.	Typing interface constituted by blocks, controlled by motor imagery through up, down, left, right imagination and eyes closed conditions.	Physionet EEG motor movement/imagery database.<br>Own dataset not available.

Table S1. Detailed notes on the 84 reviewed papers. The notes are organized to provide a clear reference to the papers and to follow the core section of the review (Section 5), analyzing (i) field of applications, (ii) employed technologies, (iii) signal processing and analysis methodologies, (iv) BCI feedback, and (v) dataset information.

**Reference**	**Application**	**Technologies (wireless, electrode type, storage, communication protocol)**		**Signal processing & analyses**			**Feedback**	**Dataset**
		**EEG related**	**other**	**pre-processing**	**feature engineering**	**classification and/or other analyses**		
Zhang, Y., Zhang, X., Sun, H., Fan, Z., & Zhong, X. (2019). Portable brain-computer interface based on novel convolutional neural network. Computers in biology and medicine, 107, 248-256.	Design of a portable dry-electrode and wireless BCI to use in MI paradigms (left and right hand MI).	Custom EEG portable headset integrated to headphones, with dry electrodes (copper probe like comb, gold surface), ADC (ADS1298, Texas Instruments) and wireless microprocessor (Nordic nRF51822). The electrodes corresponded to C3, C4, O1, and O2. Reference and ground were designed to be on the left and right earlobes, respectively.	Data trained for classification on Nvidia GTX1060 GPU with CUDA9. Bluetooth for communication.	Software used: MATLAB. Denoising: band-pass (0.5 - 100 Hz) IIR filter and notch filter (50 Hz).	Feature extraction through wavelet packet decomposition. Feature selection: features involving only alpha and beta rhythms.	Analyses of alpha waves for recording system validation: comparison between the custom system and the SynAmps2 system (Neuroscan Co., Ltd) revealed similar results. CNN (5-layers) for classification: Adam optimizer, minimization of the cross entropy loss function	Visual feedback on screen in respect to correct imagination (at a second session after training).	Not available
Zhou, B., Wu, X., Lv, Z., Zhang, L., & Zhang, C. (2015, December). Independent component analysis combined with compressed sensing for EEG compression in BCI. In 2015 10th International Conference on Information, Communications and Signal Processing (ICICS) (pp. 1-4). IEEE.	Propose EEG compression technique to reduce the amount of transmission data.	8 channels: Fp1, Fp2, C3, Cz, C4, O3, Oz, O4 sampled at 250 Hz.	NA	Bandpass filtered (8 - 30 Hz), covering mu and beta rhythm bands.	Data compression implemented by ICA combined with compressed sensing.	The proposed EEG compression scheme is compared with the traditional compressed sensing compression scheme. The two system schemes have the similar classification accuracy. The amount of transmission data can be reduced by 75%.	None	Not available
Zhuang, J., Geng, K., & Yin, G. (2019). Ensemble learning based brain–computer interface system for ground vehicle control. IEEE Transactions on Systems, Man, and Cybernetics: Systems, 51 (9), 5392-5404.	Ground vehicle control.	Emotiv Epoc.	EEG signals from the testing subject are transmitted to the PC via Bluetooth.	Bandpass filter (7 - 40 Hz). Wavelet and canonical correlation analysis used for artifact removal and improving signal-to-noise ratio.	CSP.	4 class MI classification with SVM, CNN, ensemble learning.	None	Not available
Zich, C., De Vos, M., Kranczioch, C., & Debener, S. (2015). Wireless EEG with individualized channel layout enables efficient motor imagery training. Clinical Neurophysiology, 126(4), 698-710.	MI neurofeedback can be realized with a bipolar channel pair. To reduce the number of channels to two a longitudinal study was performed over four days of MI practice.¬ On days 2–4 practice took place in an everyday environment.	Day 1 training with high-density EEG system (94 electrodes), day 2-4: modified Emotiv (easy cap with 5 electrodes, channels C3, C4 and FCz).	Also record EMG. Day 1: lab environment, Days 2-4: noisy office room. Data recorded with OpenViBE.	Bandpass filtered (8 - 30 Hz).	CSP and ICA channel selection for 94-channel EEG recordings from the first day.	Data analysis offline using EEGLAB and BCILAB. OpenViBE used to calculate the single-trial classification both online and offline. ERD analyses is also provided.	Neurofeedback.	Not available
Zich, C., Schweinitz, C., Debener, S., & Kranczioch, C. (2015, October). Multimodal evaluation of motor imagery training supported by mobile EEG at home: A case report. In 2015 IEEE International Conference on Systems, Man, and Cybernetics (pp. 3181-3186). IEEE.	Explore functional and structural changes using a multimodal framework after four weeks of MI neurofeedback training.	High-density EEG and electromyogram (EMG) data were acquired with sintered Ag/AgCl electrodes and BrainAmp amplifiers. Low-density EEG data were recorded at the participant's home from 24 Ag/AgCl electrodes embedded into an elastic cap (Easycap, Herrsching, Germany) using a mobile EEG amplifier (mBrainTrain, Belgrade, Serbia).	Record also MRI data.	Bandpass filter (8 - 30 Hz), ICA for eye blinking removal.	CSP.	LDA analysis and classification.	Online neurofeedback.	Not available
